# Stabilisation of waterlogged archaeological wood: the application of structured-light 3D scanning and micro computed tomography for analysing dimensional changes

**DOI:** 10.1186/s40494-022-00686-6

**Published:** 2022-05-12

**Authors:** Jörg Stelzner, Ingrid Stelzner, Jorge Martinez-Garcia, Damian Gwerder, Markus Wittköpper, Waldemar Muskalla, Anja Cramer, Guido Heinz, Markus Egg, Philipp Schuetz

**Affiliations:** 1 Römisch-Germanisches Zentralmuseum, Leibniz Research Institute for Archaeology, Ernst Ludwig Platz 2, 55116 Mainz, Germany; 2 Lucerne University of Applied Sciences and Arts-School of Engineering and Architecture, Technikumstrasse 21, Horw, Switzerland

**Keywords:** Conservation, Waterlogged archaeological wood, Volume, Shrinkage, Computed tomography, Structured-light 3D scanning

## Abstract

Cultural heritage objects made of wood can be preserved under waterlogged conditions for many years, where decay is slowed down and the wood structure is more or less completely filled with water. Depending on the degree of degradation, finds may collapse and shrink when they are allowed to dry in an uncontrolled manner after excavation, leading to total loss of the object and its information. Conservation measures are taken to prevent damage of objects and dimensional stability is an important criterion. In this study, structured-light 3D scanning and micro-computed tomography were used to analyse the dimensional stability of wood after conservation, as well as its long-term stability. 83 samples from a reference collection established between 2008 and 2011 allowed this comparative study of the most common conservation methods at that time. The effects of conservation methods using alcohol-ether resin, melamine-formaldehyde (Kauramin 800^®^), lactitol/trehalose, saccharose, and silicone oil on dimensional stability were investigated. In addition, different polyethylene glycol (PEG) treatments with subsequent freeze-drying were also investigated: one-stage with PEG 2000, two-stage with PEG 400 and PEG 4000 and three-stage with PEG 400, PEG 1500 and PEG 4000. The data received from analyses of both volume and surface gave detailed information about the success of each conservation method. Attempts were made to quantify the damage patterns, specifically shrinkage, collapse, and cracks. While PEG and freeze-drying, alcohol-ether-resin, as well as the Kauramin 800^®^ method gave the best results, analysis also highlighted the failures of each method.

## Introduction

Wood can be preserved in waterlogged anoxic environments for thousands of years. There, only a few microorganisms such as bacteria and fungi that use wood as a nutrient are viable in an environment with a low oxygen level [1–7]. During burial, microorganisms have had time to degrade the wood by actually consuming the material from it so that physical weakening of the structure has occurred. Water swells the wooden structure and fills the pore spaces—the capillaries and the microcapillaries. As the cell wall suffers from material loss more water will fill up the internal voids. Though, the moisture content of decayed wood is raised. The maximum moisture content is related to the state of preservation of wood and is a universally used indicator [8–10].

Depending on its condition, waterlogged archaeological wood will change its dimensions in two stages upon drying, due to collapse and shrinkage [11]. Above the fibre saturation point, cell cavities or lumina will collapse meaning irregular distortion unforeseeable in extent and distribution. The cause of collapse is capillary tension exerting compressive forces on the cell wall. As a result, the considerably weakened cell walls inevitably collapse [8, 12, 13]. Because of its heterogeneous state of preservation, there will be stresses developed in the object. In particular, the decayed shelf will dry first while the core is still wet [14].

Below the fibre saturation point, the cell walls will shrink [8, 15, 16]. The wood will then contract to a minimum of the original dimensions. Shrinkage in the tangential direction is generally more severe than radial and longitudinal shrinkage. But volumetric shrinkage is directly proportional to the water content of archaeological wood [10, 14].

There are several criteria to assess the effectiveness of conservation methods [14, 16–18]. One of the main criteria is to prevent the wood from shrinkage and to stabilise the volume of the object. The stabilisation involves the preservation of the shape of the object, which contains information such as the manufacturing technique and the function of the object [19]. The dimensions of the actual but swollen waterlogged state should be preserved and intervention should be kept to a minimum [20].

To avoid shrinkage and collapse of waterlogged wood upon drying is the main challenge for the conservation of archaeological wet finds [21, 22]. The damaging effect of air-drying and the question of how to solve this problem has already been noted in the nineteenth century as being not an easy task [23, 24]. Since then, a variety of methods and conservation agents have been tested in conservation [16, 25–33]. The heterogeneous material, the different types of wood and especially the widely varying states of preservation make it difficult to assess the success of a conservation method, and studies to compare different conservation methods have been made since the beginning of wood conservation [8, 18].

To check the dimensional stabilisation of a method, the condition after the conservation is compared with the condition before conservation. In conservation science, several measurement methods have been applied to assess methods by comparing the dimensions before and after conservation: The extent of dimensional changes was evaluated on thin sections under the microscope [13] or on wooden samples with exact dimensions [16, 18, 19, 34–42]. The surfaces were also compared by drawing the outline of objects [43]. To assess the anisotropic character of archaeological wooden objects during drying, samples were cut in accordance with the specific alignments [17, 44] or stainless pins were introduced into the wood [15, 16, 34, 45–51]. In recent times, also optical 3D measurement methods are used in laboratory studies [45–47, 49, 50, 52] and on shipwrecks [53].

The aim of this study is to assess the dimensional stabilisation of a number of established and most commonly used conservation methods on larger sample series consisting of different wood species. The research in this study focuses on the volume changes after conservation using structured-light 3D scanning. In addition, the sample material studied allowed to investigate the volume stability of the conservation methods after 10 years.

Normally, the evaluation of the dimensional stability of conservation methods is limited to the outer surface of the wood. Micro-computed tomography (µCT) offers a non-destructive technology for the visualisation of the structures inside. Until now there are only a few cases that have also considered changes inside wood after conservation using tomographic methods [52, 54–62]. These investigations have shown that shrinkage of the wood can also result in cavities inside. In addition to cavities, cracks in the structure can also be detected [62]. However, it is the overall structure that provides information on whether an object has been stabilised. For this reason, the samples in this study were also examined by µCT after their conservation, which allowed internal defects such as cell collapse and cracks to be taken into account when evaluating the conservation result.

## Materials and methods

### Samples

**Fig. 1:**
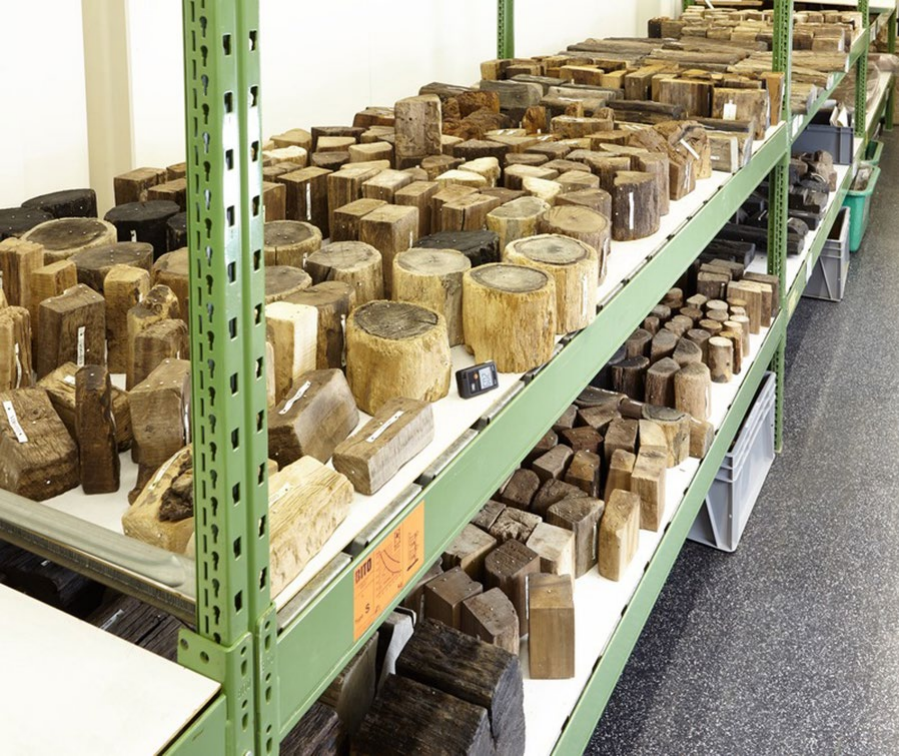
Samples of the KUR collection at the RGZM

The material under investigation is a reference collection held at the Römisch-Germanisches Zentralmuseum (RGZM), Leibniz Research Institute for Archaeology. In a research project that was performed from 2008 to 2011 the most established conservation methods were investigated where approximately 800 samples were conserved at different Institutions with their standard methods (Table 1, Fig. 1). A detailed overview of the conservation methods and the samples is given on the project homepage [45]. The methods employed were alcohol-ether-resin [18], melamine-formaldehyde (Kauramin 800^®^) [63], lactitol/trehalose [64], saccharose [26], silicone oil [65] and polyethylene glycol (PEG) with subsequent freeze-drying. PEG treatment followed either a one-stage process with PEG 2000 [66], two-stages with PEG 400 and PEG 4000 [67] or three-stages with PEG 400, PEG 1500 and PEG 4000 [45].

**Table 1:** Overview of conservation methods

Conservation method	Institutions and short descriptions of the methods
Alcohol-ether-resin (AlEt)	Institution: Schweizerisches Nationalmuseum, Zürich, Switzerland Treatment: Exchange of water with ethanol. Exchange of ethanol with diethyl ether. Soaking of wood with diethyl ether in resin-diethyl solution. Drying by evaporation of the diethyl ether in the vacuum vessel. Application of surface protection 3% Paraloid B72 solution in acetone Impregnation solution: 70.7% diethyl ether, 16.1% dammar resin, 6.4% rosin, 3.2% dienol D102, 3.2% rhizinus oil, 0.4% PEG 400
Kauramin 800^®^ (K800)	Institution: Römisch-Germanisches Zentralmuseum, Mainz, Germany Treatment: Bath impregnation at room temperature. Replacement of the solution when early polymerisation occurs. Curing of the impregnated wood in the heating cabinet at 60 °C. Afterwards slow, controlled air-drying. Dip in linseed oil varnish Impregnation solution: 25% Kauramin 800^®^ solution (72 L resin + 210 L deionised water, 3.6 L urea, 7.2 L triethylene glycol)
Lactitol/trehalose (LaTr)	Institution: Brandenburgisches Landesamt für Denkmalpflege, Zossen, Germany Treatment: Starting with 30% concentration. Increasing monthly in 10% steps up to 70%. Bath temperature 55 °C. After removal from the bath, the surfaces were dusted with crystalline lactitol monohydrate and dried in a heating oven over a period of 1 week. After drying, the surface was cleaned by dabbing with damp cloths Impregnation solution: lactitol/trehalose solution (9:1) 30–70%. Addition of biocide if necessary (0,1% Bioban 404)
Polyethylene glycol (PEG 2000) one-step and freeze-drying (PEG1)	Institution: Nationalmuseet, Copenhagen, Denmark Treatment: Starting with 10% PEG 2000 solution. Increasing the concentration up to 40% at room temperature. Freeze-drying in cooled chamber (approx. − 30 °C). Removal of excess PEG from the surface with a soft brush and ethanol. Subsequent surface stabilisation with 25% PEG 2000 solution in ethanol Impregnation solution: PEG 2000, 10–40% solution with tap water
Polyethylene glycol (PEG 400 and 4000) two-step and freeze-drying (PEG2)	Institution: Brandenburgisches Landesamt für Denkmalpflege, Zossen, Germany Treatment: Soaking in demineralised water. Starting with 5% PEG 400 solution. Raising the concentration in 5% steps. At its calculated final concentration, it was kept constant. Then the increase of PEG 4000 solution was continued in 5% steps up to its final concentration. Precooling of the wood to 5 °C then deep-freezing to − 25 to − 35 °C, freeze-drying in cooled chamber (approx. − 30 °C) Impregnation solution: PEG-solution in demineralised water (PEG 400 and PEG 4000) was adapted according to the condition of the wood (PEGcon)
Polyethylene glycol (PEG 400, 1500 and 4000) three-step and freeze-drying (PEG3)	Institution: Archäologische Staatssammlung, Munich, Germany Treatment: Soaking in demineralised water. Starting with 11% increasing to 15% PEG 400 solution at room temperature. 16% increasing to 20.5% PEG 1500 solution at 40 °C. 20.5% increasing to 27.5% PEG 4000 solution at 40 °C. Washing of the wood and wrapping in cellulose tissues. Intermediate storage in freezer (− 25 to − 35 °C) until freeze-drying. Subsequent freeze-drying in a cooled chamber (approx. − 30 °C). Excess of PEG was removed with a brush and ethanol Impregnation solution: PEG-solution in demineralised water: 15% PEG 400, 6.5% PEG 1500, 7% PEG 4000
Saccharose (Sac)	Institution: Sächsische Landesamt für Archäologie, Dresden, Germany Treatment: Concentrated the solution in 10% steps, from 10% up to 60% sugar solution at room temperature. Slow, controlled air-drying in microperforated bags. Removal of crystallised sugar residues from the surface with damp sponge Impregnation solution: Aqueous saccharose solution 10–60%. If necessary biocide addition composed of 0.6%, sodium benzoate (E211), 0.5% Parmetol K40, 0.5% Quartasept Plus and 0.02% Tallofin OT
Silicone oil (Sil)	Institution: Texas University, Texas, USA Treatment: Exchange of water with ethanol. Exchange of ethanol with acetone. Placing the still dripping wet acetone-impregnated samples in impregnation solution under normal atmospheric conditions. Triggering the polymerisation of the impregnation solution by gaseous catalyst: DBTDA (dibutyl diacetate) Impregnation solution: 80% silicone oil (SFD1 (66%) + SFD5 (34%) − silanol functional polydimethylsiloxanes “PDMS”) and 20% crosslinker MTMS (methyltrimethoxysilane)

The samples were taken from archaeological objects that were collected from different sites. The objects were made of different types of wood and have different degrees of degradation, which were divided into the samples. The degree of degradation was determined by the maximum water content (U_max_). The mass of the waterlogged wood was determined without vacuum impregnation with water before measuring. U_max_ is defined as the water present in the sample compared to the absolute dry wood substance [2, 10]:

1 $$Umax= \frac{mass\, wet\, wood-mass \,oven \,dried \,wood}{mass\, oven \,dried\, wood}\, [\%]$$

Values of U_max_ have been classified by de Jong [51]. The numbers correspond to the water content ranges of the samples: (1) U_max_ > 400%, (2) U_max_ 185–400%, and (3) U_max_ < 185%. The maximum water content was determined destructively for the control samples to be air-dried, and non-destructively for the samples to be conserved [45, 67]. The density of the cell wall substance is usually assumed to be 1.5 $\frac{g}{{cm}^{3}}$ [10, 68]. The water content is proportional to the void volume in the wood and inversely proportional to the basic or conventional density (BD, standard ISO 13061) of the wood in question (amount of wood substance per volume, g/cm^3^) [2, 69, 70]:

2 $$BD=\frac{100}{\left(66.7+{U}_{max}\right)} \left[\frac{g}{{cm}^{3}}\right]$$

To get an impression of how much wood has been decayed, the residual basic density (RBD) was calculated, in percent, as the ratio between the measured density of the archaeological material and the average basic density for non-degraded wood of the same species [10, 69], as derived from the literature [14, 71]:

3 $$RBD=\frac{BD}{BD (fresh \,wood)}\bullet 100\left[\%\right]$$

The non-destructive determination of the condition of the wood does not always lead to reliable results, due to both mineral inclusions and inclusions of air in intact fibres with non-degraded pits and cell-walls. Therefore, the average value from all samples was taken (Appendix A).

**Fig. 2:**
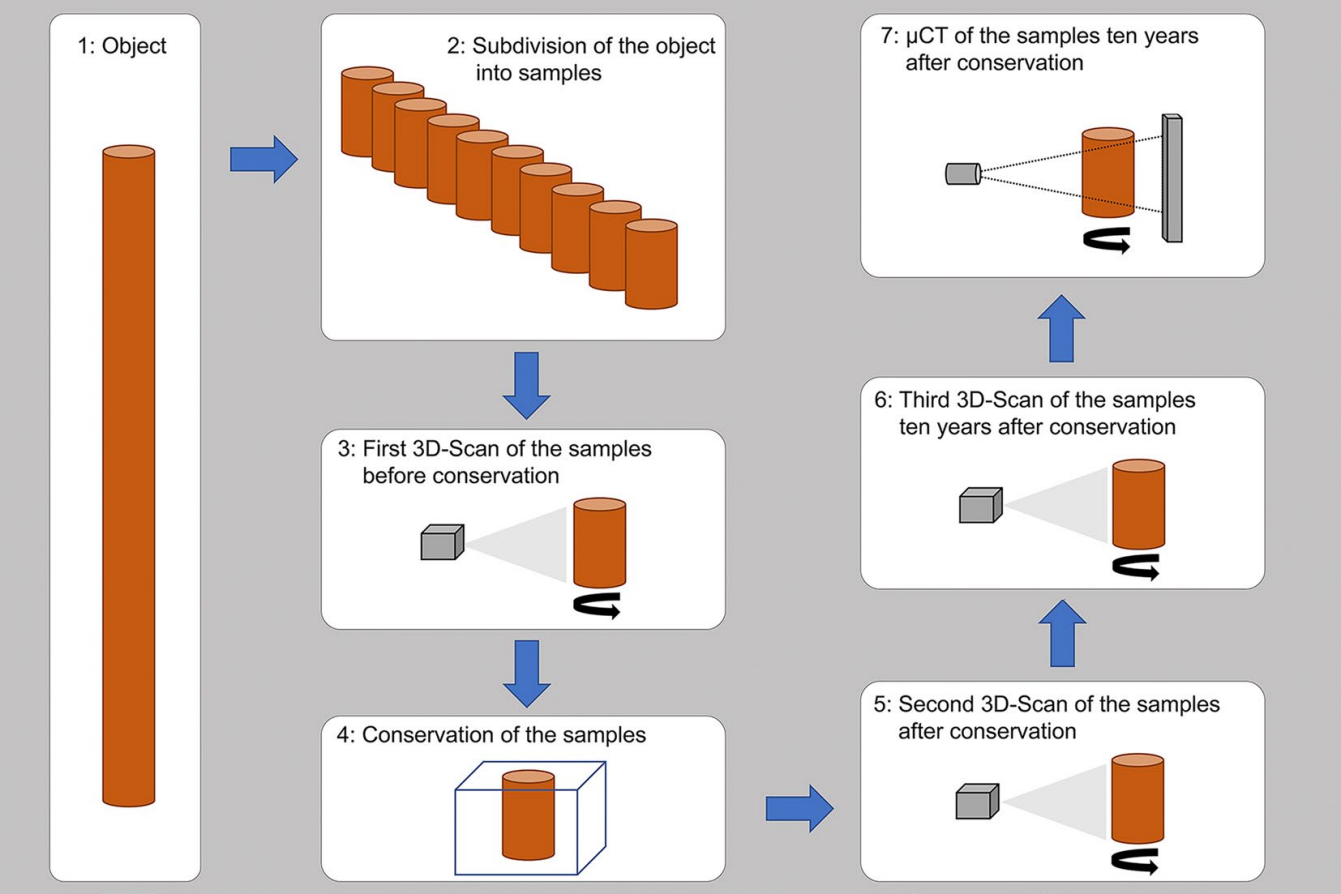
Graphical overview of the experimental procedure: sampling, conservation, measurements with structured-light 3D scanning and µCT

This systematic collection of samples provides a unique chance to compare the structural differences of the conserved wood [43, 46]. Each test series of the collection includes samples derived from the same object (same wood species and finding place, similar state of preservation) that were conserved with the different treatments. Due to the different sizes of the objects, the number of samples varied. Therefore, not all test series contain all conservation methods. For the volume analyses in this study, the ten largest test series of the collection were selected in order to cover a high variety of methods and wood genera. Sample series of different wood species were investigated: three from oak (Oa1, Oa2 and Oa3), two from fir (Fi1 and Fi2) and one from alder (Al1), ash (As1), beech (Be1), pine (Pi1) and spruce (Sp1). 83 samples were analysed (Appendix A). Figure 2 gives an overview of the sampling and measurement methods of this research.

### Structured-light 3D scanning

From 2008 onwards, the wet samples were captured before conservation and in dry condition after conservation using a structured-light 3D scanner. The capturing device was an ATOS III Rev. 01 scanner from GOM (a ZEISS company) with a field of view of 500 mm × 500 mm × 500 mm and a point distance of 0.25 mm. After data capture, all scans were processed in the ATOS Professional 2016 Software using same parameters by employing Python scripts to control the workflow and obtain a reduced 3D mesh with a closed surface. To analyse volume changes after 10 years the conserved samples were captured again in 2020. Since the same sensor was no longer available, a successor model was used. The ATOS III Rev.02 Triple Scan from GOM (a ZEISS company) with a field of view of 320 mm × 240 mm × 240 mm and a point distance of 0.10 mm was used. The processing of scans results in 3D meshes of the samples.

### Micro-computed tomography

Analysis of the condition of the wood structure inside the samples about 10 years after their original conservation was carried out at Lucerne University of Applied Sciences and Arts with a µCT-system. The tomographic analysis was performed on the in-house laboratory XCT system (Diondo d_2_, Germany). An optimised setup and acquisition protocol for the µCT measurements was developed for conserved wood. The measurements were conducted by setting the X-ray source (XWT-225 TCHE+ from X-ray works, Garbsen, Germany) in high power mode and choosing an operation voltage of 120 kV and a filament current of 167 μA with a 1 mm aluminum filter. The wood samples were mounted in a sample holder and placed in the sample chamber. The sample was rotated 360° in continuous mode during the acquisition. The radiographical projections were recorded with a 4343 DX-I X-ray detector (Varex, Salt Lake City, USA), with a pixel size of 139 μm. The distance between the X-ray source and the sample was between 160 and 250 mm and the distance between the X-ray source and the detector was 860 mm, giving a magnification between 3.4 and 5.4 and a nominal voxel size between 27 and 44 μm. A total of 3000 projection images were acquired during the sample rotation of 360°. The resulting projections were converted into a 3D image stack of approx. 3000 × 3000 × 3000 voxels using the CERA reconstruction software based on the filtered back projection Feldkamp algorithm [72] from Siemens. The achieved resolution of the µCT measurements depends on the size of the samples [73]. Appendix A gives an overview of the samples, their size and the achieved resolution of the µCT measurements. Image cross-sections and 3D renderings of the wood were visualised in VGStudioMax3.4© software.

### Surface and volume determination and calculation of the dimensional changes

To get information about the surface and the volume of the samples measured with µCT the data was analyzed with VGStudioMax3.4. To obtain values of the wood volume concerning inner cracks and collapse, surface determination of the very heterogeneous material was done manually for each sample with the different segmentation tools of the software. Afterwards the surface and volume values of the segmented µCT data and that of the structured-light 3D scanning were calculated with the same software.

The evaluation of the conservation methods was done by determining the dimensional stability on the basis of the volume data that was derived from the surface of the whole sample. The values of the individual wood anatomical directions (tangential, radial and longitudinal) can be taken from the database [45].

To evaluate the volume changes in the measurement, data from structured-light 3D scanning was used to calculate the shrinkage (S) from the volume of the samples before (V_wet_) and after (V_dry_) conservation [74–77]:

4 $$S=\frac{{V}_{wet}-{V}_{dry}}{{V}_{wet}} \times 100\, [\%]$$

The anti-shrink efficiency (ASE) was determined from the shrinkage of a non-conserved control sample (S_o_) and the shrinkage of the conserved sample (S_con_) [15, 16, 78]:

5 $$ASE=\frac{{S}_{0}-{S}_{con}}{{S}_{0}} \times 100\, [\%]$$

100% ASE means a very good conservation has been achieved, whereas an ASE of 0% indicates a result equal to that accomplished by air-drying. An ASE of 75% seems to be acceptable [15, 16]. By using ASE, a statistical evaluation and comparison of conservation results is possible.

To probe the volume stability of conserved archaeological wood over time and the dimensional stability of the wood volume with respect to the inner structure, the values of the whole samples from structured-light 3D scanning and µCT were used. Analogous to the shrinkage after conservation analysis (Eq. 4), the volume change of the samples after 10 years was calculated from the volume after conservation and the volume 10 years after conservation. Similarly, the volume change concerning the inner structures of the wood was calculated from the volume of the structured-light 3D scanning 10 years after conservation and the volume of the µCT-data after 10 years. Surface changes (A_O_) were calculated additionally from the values of structured-light 3D scanning and µCT after 10 years:

6 $${A}_{O} change=\frac{{{A}_{O\, \mu CT}}-{{A}_{O\, scan}}}{{{A}_{O\, scan}}} \times 100 [\%]$$

To get an impression of the size and shape of the cavities inside the samples the volumes and surface areas were determined by the difference between structured-light 3D scanning and µCT data. From these values the sphericity ѱ was calculated:

7 $$\uppsi = \frac{{\sqrt[3]{{36\pi V^{2} }}}}{{A_{O} }}$$

The sphericity ѱ relates the shape of a body based on its volume (V) and its surface (A_O_) to the smallest possible surface of a sphere of the same volume [79]. The value of the sphericity ѱ for a sphere is 1. The lower a value of sphericity ѱ is for a shape, the larger the surface area is compared to a sphere.

## Results and discussion

### Volume changes

**Fig. 3:**
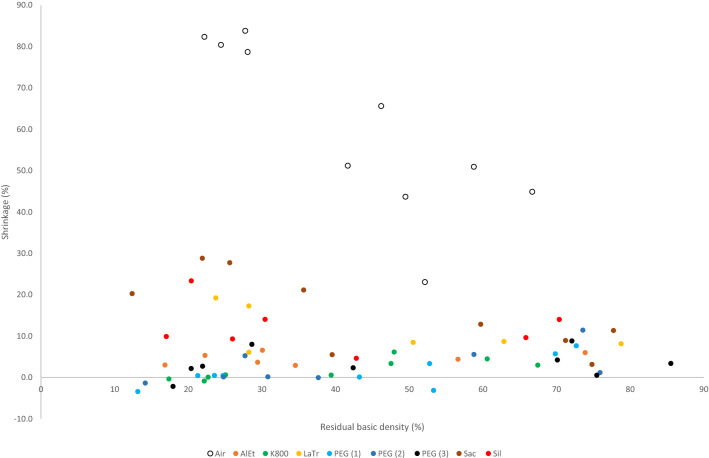
Loss of volume after air-drying untreated samples and conserved ones in relation to the condition of the wood (residual basic density in %)

Appendix B shows the values for the volumes of the structured-light 3D scans before and after conservation. The measurements show extensive shrinkage of the unconserved wood samples. In Fig. 3 the loss of volume after air-drying of the untreated wood shows a relationship with the condition of the wood which is calculated as the residual basic density in per cent (Eq. 3). There is a clear trend showing that the better the wood is preserved, the less volume change occurs during air-drying. Figure 3 also demonstrates less shrinkage of all conserved samples in comparison to the air-dried samples. There is a difference between the conservation methods concerning the samples with a higher degradation and a lower residual basic density. It is evident that the stabilisation of these samples treated with saccharose, lactitol/trehalose or silicone oil is not as sufficient as compared to the other treatments.

**Fig. 4:**
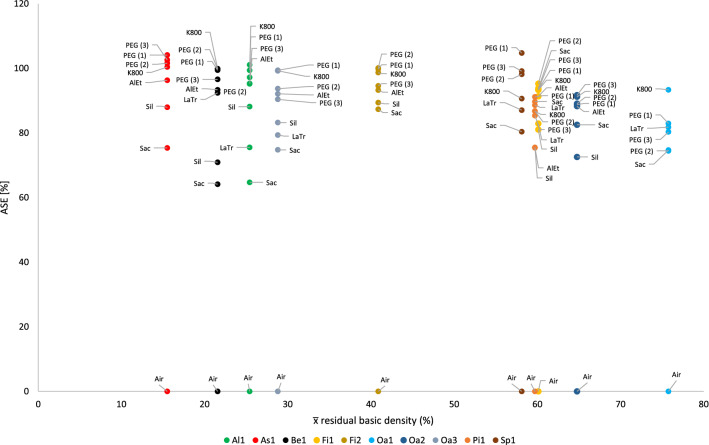
Volume stability (ASE) dependent on the average residual basic density x̅. The conservation method is given on the data point

**Fig. 5:**
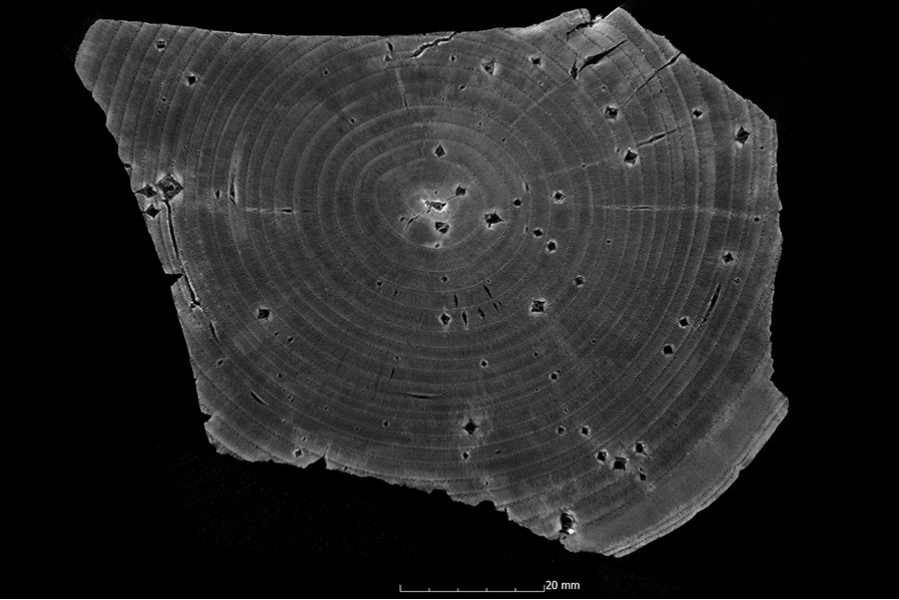
µCT cross section of the ash sample conserved with Kauramin 800^®^ (As1-K800) with one homogeneous state of preservation

**Fig. 6:**
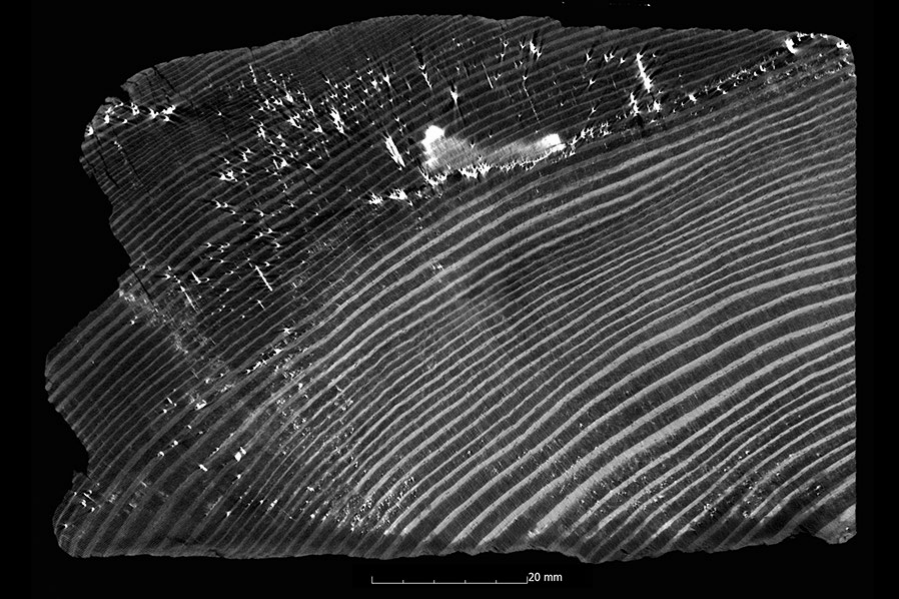
µCT cross section of the pine sample conserved with alcohol-ether-resin (Pi1-AlEt) with high degraded area left and low degraded area right

This tendency is also confirmed by the calculation of the ASE (Eq. 5). Figure 4 shows the ASE of all sample series in relation to the preservation status. For a better overview, the average value (x̅) of the residual basic density is given here for each sample series. In Fig. 4 it becomes even clearer that strongly degraded sample series are less stabilised by the conservation agents saccharose, lactitol/trehalose and silicone oil than by the other conservation agents. In addition, it also becomes obvious that the overall volume stabilisation decreases with a better state of preservation. The threshold here is a residual basic density of about 60%. This limit indicates that the preservation state 2 after de Jong [51, 80, 81] is particularly difficult to conserve due to two different states of preservation in one object [17]. The µCT data confirms that in these test series (Pi1, Fi1, Oa1 and Oa2) samples have highly degraded and low degraded areas. In contrast, the more degraded sample series show a consistent picture in the µCT data. The cross-sections of the samples As1-K800 and Pi1-AlEt show the difference between one homogeneous state of preservation (Fig. 5) and two states of preservation in one object (Fig. 6).

**Fig. 7:**
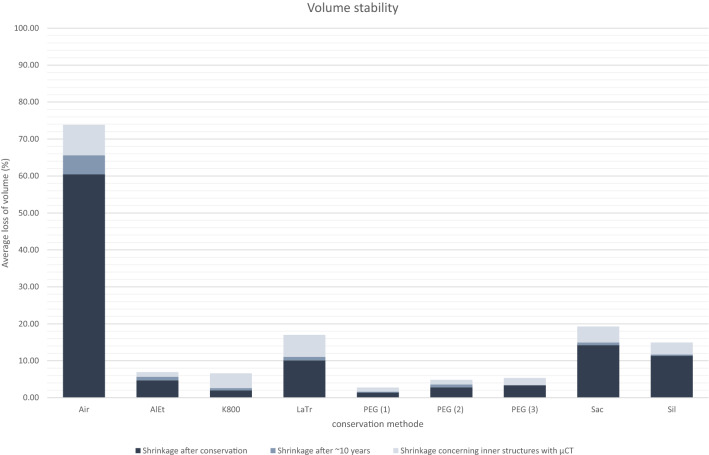
Average shrinkage of all selected samples after conservation, after 10 years and including the inner structures derived from µCT

Apart from the state of preservation, there are also considerable variances in the conservation methods in the individual test series with different wood species. All conservation methods improve stabilisation to some degree. Taking all the results into account and looking at the average of the volume changes for the different conservation methods, a general trend can be seen (Fig. 7). The best results are mostly achieved using PEG, the alcohol-ether method or Kauramin, although there are exceptions. The individual conservation methods with their deviations and special features will be discussed later in detail.

**Fig. 8:**
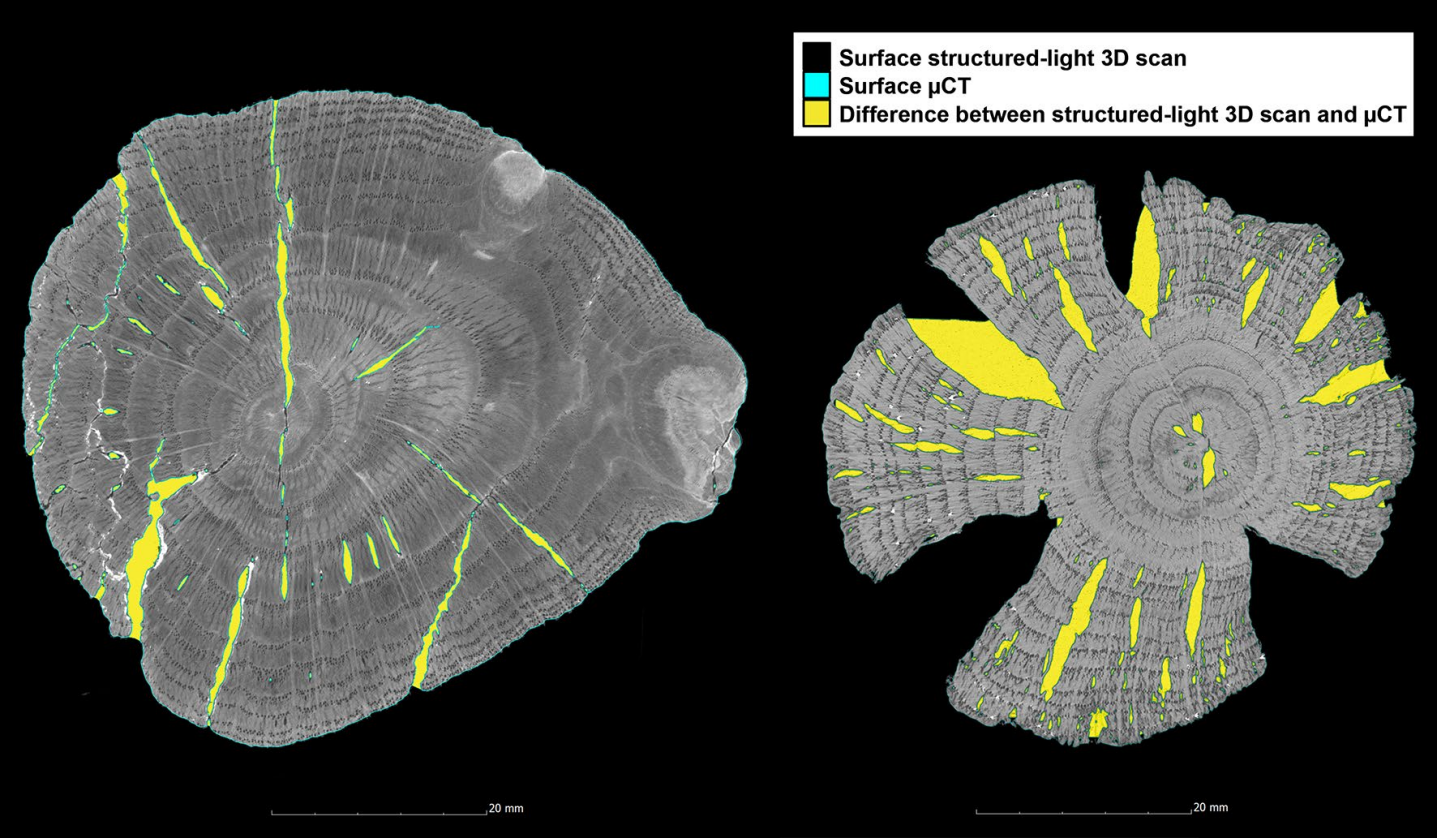
µCT cross sections of two examples (Oa3-K800 left and Oa3-Sac right) for the comparison of the surface detection with structured-light 3D scanner and µCT

Appendix C shows the values of the volume changes after 10 years. Here, it is particularly noticeable that almost all unconserved wood samples are not stable. With a few exceptions, the changes in the conserved samples are very slight and no clear trends can be identified for the individual conservation methods. All conservation methods thus seem to permanently stabilise the samples, as can be seen from the average values for volume change after 10 years (Fig. 7). In Appendix C, the internal structures are considered and the extent to which this changes the volume. Since no µCT data of the previous state is available, the values of the µCT data are compared with the values of the surface scans of the current state 10 years after conservation. Since the original condition of the interior of the samples is unknown, this is primarily a comparison of methods. It should be noted here that the µCT not only considers internal cavities, but also, in contrast to the surface scan, considers and more accurately depicts crevices and depressions (Fig. 8). Nevertheless, differences in the conservation methods become apparent when looking at the average value of the volume change inside all samples. The conservation methods with Kauramin 800^®^, saccharose, lactitol/trehalose and silicone oil seem to lead to a greater change in volume inside the samples than the other conservation methods (Fig. 7).

### Volume changes in relation to the surface

Another indicator for the stabilisation of a sample that can be obtained from surface scans and µCT are changes in surface values. Obviously, if only the outer surface is considered, it will also decrease with volume shrinkage. By comparing the surface data with the µCT data, a value for the surface inside the sample is obtained, which can provide information about cell collapse and cracks in the sample (Appendix D, Eq. 6).

**Fig. 9:**
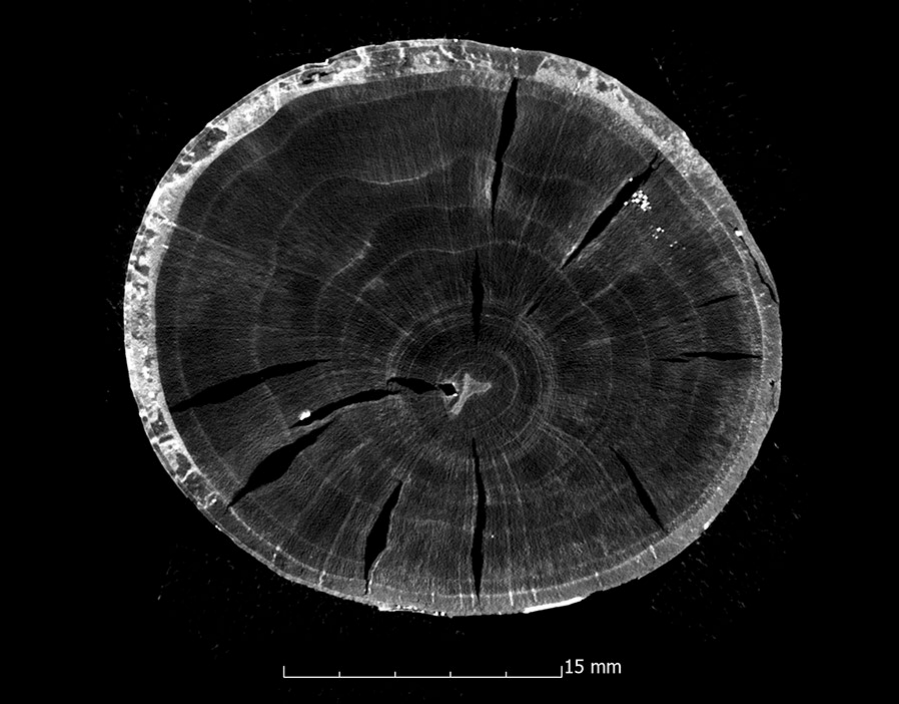
µCT cross section of the alder sample treated with alcohol-ether-resin (Al1-AlEt) with cell collapse in the wood structure

If the surface area increases in the µCT data, we are dealing with cavities inside the sample. Again, it must be considered that due to the lack of the previous condition, it cannot be clearly proven whether the cavity was already inside the wood before the conservation. In some cases, there is also some uncertainty due to deeper cracks in the surface that were not detected by the structured-light 3D scanner (Fig. 8). When looking at the µCT data, however, cell collapse is definitely evident (Fig. 9). In addition, clear cracking can be seen in the samples conserved with PEG that were freeze-dried (Fig. 14) [46, 52].

**Fig. 10:**
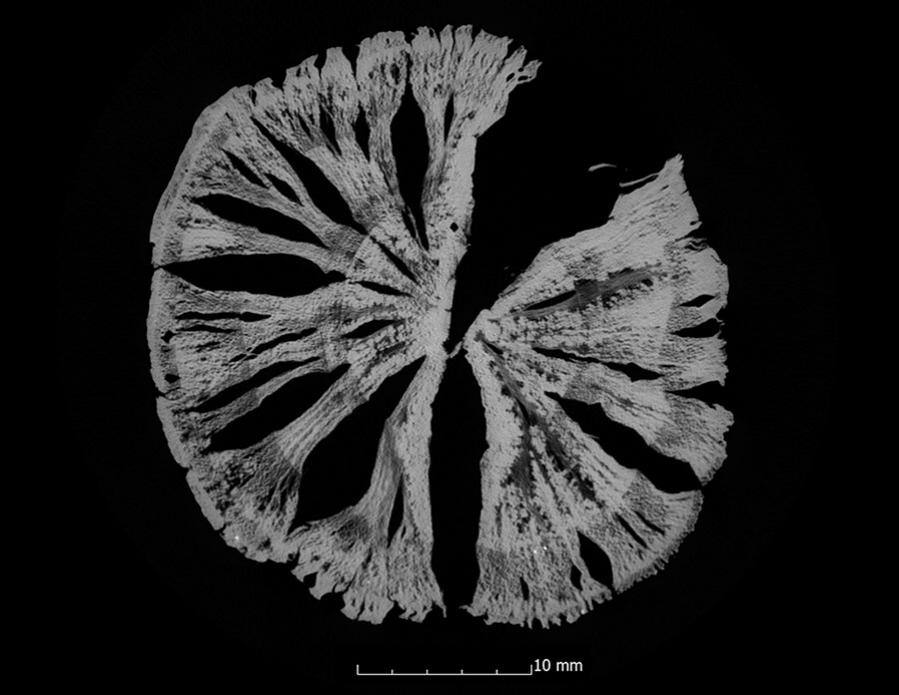
µCT cross section of the alder sample treated with lactitol/trehalose (Al1-LaTr) with cell collapse in the wood structure

The values for the enlargement of the surface and the shrinkage in percent, resulting from the comparison of surface scan and µCT, are listed in Appendix D. From the values, it is possible to see, to some extent, if collapse and cracks appear inside the wood samples. If neither the volume nor the surface have changed much, this means that the sample is also stable inside. In contrast, it can be assumed that a large surface increase combined with a large volume loss can only be explained by a significant collapse inside the sample. An example of this is the alder sample (Al1-LaTr, Fig. 10). A surface increase of 305% and a shrinkage of 16%. If only the volume has changed considerably and the surface does not change so significantly, it can be assumed that isolated, large cracks or fissures have occurred within the wood. This is the case with the oak sample (Oa2-K800), which is also shown in the cross-section of the sample (Fig. 13). In relation to the volume loss with 15%, the surface with 144% has changed far less compared to the alder sample (Al1-LaTr, Fig. 10). If, on the contrary, the surface has increased considerably with a smaller change in volume, this is a sign of many fine cracks within the wood, as has already been mentioned for the wood conserved with PEG and freeze-dried. An example of this is the ash sample (As1-PEG1), which has a relatively stable volume with a shrinkage of 1% but a large surface enlargement with a value of 218%. The fine cracks, leading to these values, can be seen in Fig. 14. Such obvious changes in the wood structure and the measured volume and surface values are discussed below individually for each conservation method studied.

**Fig. 11:**
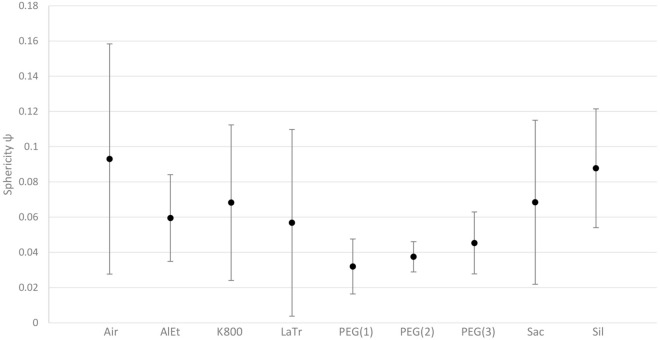
Sphericity ѱ (average values with standard deviation) of the cavities inside the samples determined for the different conservation agents from structured-light 3D scanning and µCT data

Another way to approximate the shape of these cavities inside the wood is to calculate the sphericity ѱ, which relates the shape of a body based on its volume and its surface to the smallest possible surface of a sphere of the same volume (Eq. 7) [79]. The lower this value for a shape, the larger the surface in relation to a sphere. The values determined in this way show a wide variation and are probably dependent on the conservation agent, the type of wood and the state of preservation. However, tendencies for the different conservation methods can be derived from the averaged values (Fig. 11). In contrast to the other methods, the values obtained from samples treated with PEG scatter far less. This could be an indication that a more uniform and reliable conservation result can be achieved here. Furthermore, the average values obtained from samples treated with PEG are noticeably low. The sphericity ѱ confirms the picture that fine cracks tend to form in these cases.

### Dimensional stabilisation provided by the conservation methods

#### Alcohol-ether-resin

The samples conserved with the alcohol-ether-resin show very good volume stabilisation overall. With two exceptions, the values determined for the ASE of the scans before and directly after conservation are above 90% (Appendix B). The oak sample (Oa2-AlEt) also has a very good ASE value of 88%, while the pine sample (Pi1-AlEt) having the lowest value, with an ASE of 76%. This could be due to the preservation state 2 according to de Jong [51, 80, 81] with two different levels of degradation (Fig. 6). The scans after 10 years show consistently good values for volume stability. Considering the internal structures resulting from the µCT data, the method also shows good volume stabilisation (Appendix C, Fig. 7). Only the alder sample (Al1-AlEt) shows a poor value with more than 5% additional volume loss. If the surface change of the samples preserved with alcohol-ether-resin is added, the predominantly positive overall picture is reinforced (Appendix D). The low surface increase when considering the internal structures on the basis of the µCT, together with the simultaneous low volume loss, indicates that there was little collapse or crack formation in the interior of the specimens, which is also confirmed by the cross sections of the samples in µCT data. An exception is again the alder sample (Al1-AlEt), which shows a comparatively high value of 137% surface increase, which in connection with the volume loss indicates an increased cell collapse inside the sample. This is supported by the observation of the µCT data (Fig. 9). It should be noted here that in this series, poorer values are observed overall in comparison with all conservation methods, which may be explained by the fact that cavities were already present in the wood before conservation. This positive assessment is also confirmed in previous studies [18] especially for broadleaved woods [82].

#### Kauramin 800^®^

**Fig. 12:**
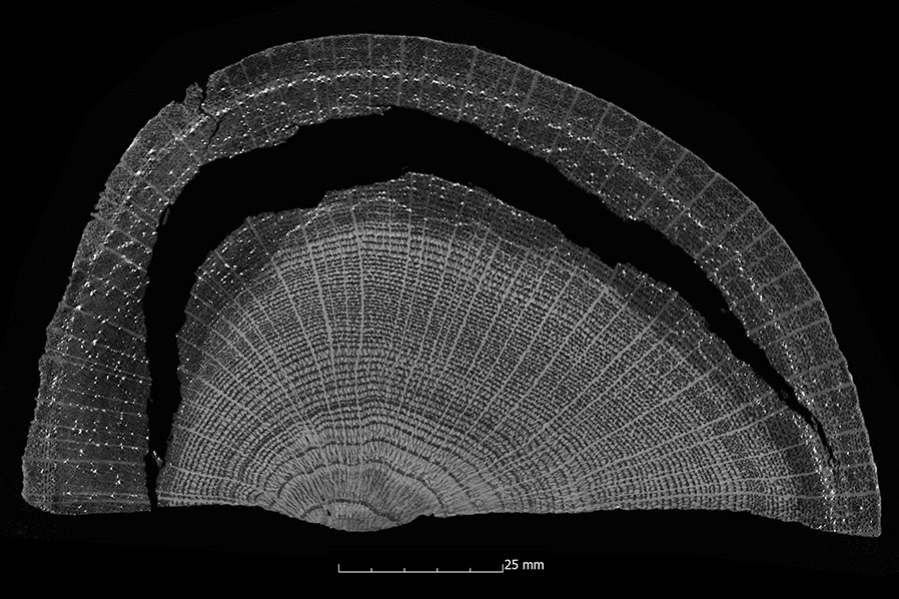
µCT cross section of the oak sample treated with Kauramin 800^®^ (Oa1-K800) with a large crack in the wood structure

**Fig. 13:**
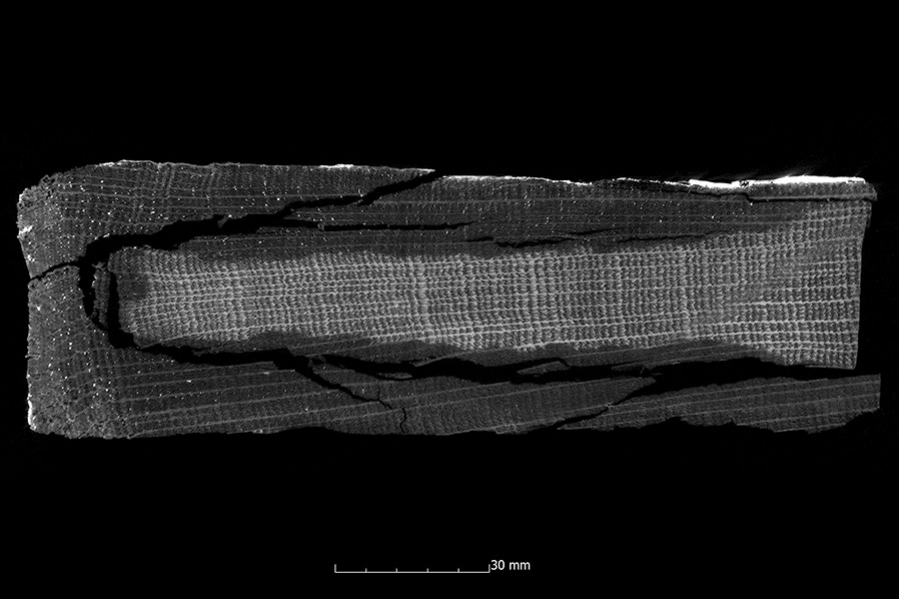
µCT cross section of the oak sample treated with Kauramin 800^®^ (Oa2-K800) with large cracks in the wood structure

The samples conserved with Kauramin 800^®^ also show mainly very good results. With two exceptions, the ASE directly after conservation shows values above 90% (Appendix B). Five of the ten samples even show an ASE of 99% to 101%. The samples with lower values are the pine sample (Pi1-K800) with an ASE of 87% and the oak sample (Oa1-K800) with an ASE of 88%. The pine sample (Pi1-K800) comes from the same sample series as the previously mentioned pine sample (Pi1-AlEt), which, with two different areas of degradation (de Jong: 2), also has the worst value of the alcohol-ether-resin. Volume stability is guaranteed for Kauramin 800^®^ even after 10 years. A somewhat different picture of the method emerges when looking at the µCT data. While the positive results for most samples are confirmed by a small additional volume loss, a different picture emerges for the oak samples (Oa1-K800 and Oa2-K800) with two different states of preservation (de Jong: 2, Appendix C). The additional volume loss of 10% and 15%, respectively, is also significantly reflected in the overall results of the method (Fig. 7). It is striking that this volume loss is caused, in both cases, by large gaps that run along the two different maintenance states in the wood (Figs. 12 and 13). The low values of surface area increase in relation to volume loss confirm here that large fissures are involved (Appendix D). Overall, the samples preserved with Kauramin 800^®^ show greater surface area increase than the samples preserved with alcohol-ether-resin, suggesting that cell collapse and cracking have increased in these. This may be due to the fact that it is too stiff and is not easy to deform. This has been criticised in the literature regarding previous methods using melamine resins [18]. It has also been described that Kauramin 800^®^ stabilises highly degraded wood very well, while well-preserved wood is stabilised more poorly. This could be due to poorer penetration of the amino resin prepolymer, resulting in collapse and shrinkage of the well-preserved wood [17]. This becomes obvious in samples with two different states of preservation.

**Fig. 14:**
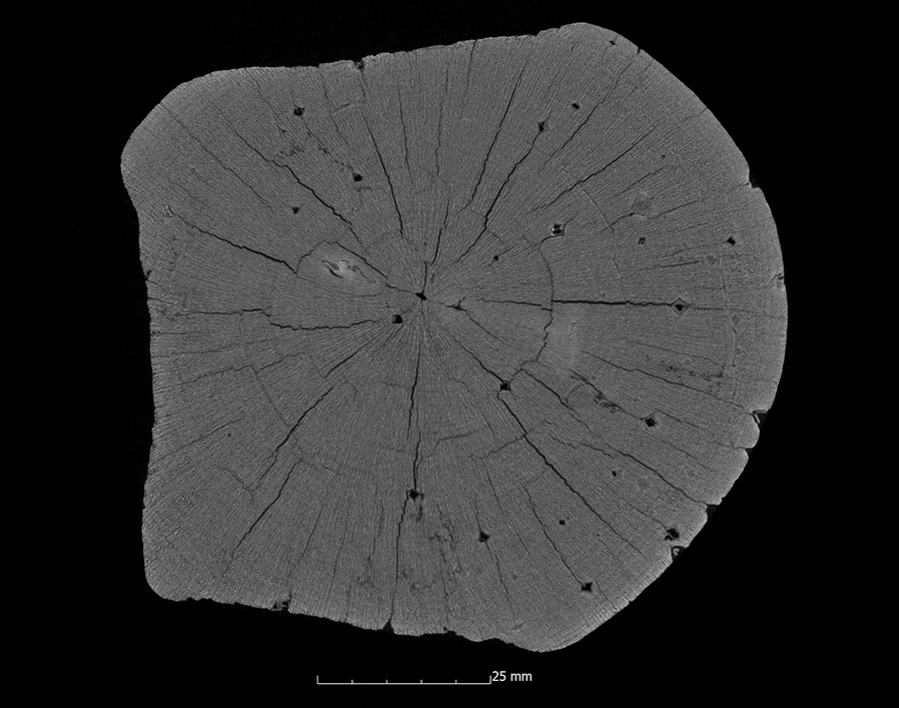
µCT cross section of the ash sample treated with PEG 2000 and freeze dried (As1-PEG1) with cracks in the wood structure

#### Lactitol/trehalose

The samples conserved with lactitol/trehalose were inferior to the previously mentioned methods. The ASE of the samples directly after conservation is between 76% and 92%, whereby only the beech sample (Be1-LaTr) with an ASE of 92% has a value above 90% (Appendix B). A change in volume after ten years is also not observed in the woods conserved with lactitol/trehalose. Looking at the internal structures based on the µCT of the samples preserved with lactitol/trehalose, this also confirms poor volume stabilisation (Appendix C, Fig. 7). In particular, the alder sample (Al1-LaTr) and the oak sample (Oa3-LaTr) have poor values with 16% and 14% additional volume loss, respectively. If one adds to the volume loss the strong surface change in these samples (Appendix D), this indicates that considerable collapse has occurred inside these samples. Figure 10 shows a cross-sectional image of the µCT, which illustrate the collapse of the wood structure.

#### Polyethylene glycol (PEG 2000) one-step and freeze-drying

The samples conserved with PEG 2000 and then freeze-dried show predominantly very good volume stabilisation (Appendix B). Except for two samples, the ASE values are above 90%. In six of the ten samples, the ASE is between 99% and 105%. The samples with the lowest values are the oak sample (Oa1-PEG1) with a value of 83% and the oak sample (Oa2-PEG1) with a value of 89%. The scans after 10 years also show consistently high values of volume stability for the samples conserved with PEG 2000. Similarly, the µCT data confirm good volume stabilisation inside the samples (Fig. 7). With just over 2% additional shrinkage inside the samples, the alder sample (Al1-PEG1) and one of the two fir samples (Fi2-PEG1) have the highest values (Appendix C). If the surface change is also considered, it is noticeable that these two samples also exhibit a large surface enlargement with values above 200%. With the exception of the first fir sample (Fi1-PEG1), such a surface increase is observed in all samples preserved with PEG 2000 and freeze-drying (Appendix D), which can be explained by the formation of cracks. Figure 14 shows these cracks, for example, in the ash sample (As1-PEG1). With the exception of the fir sample (Fi1-PEG1), these cracks occur in all samples conserved with PEG 2000. The first step in the freeze-drying process is the freezing of the impregnated wood. Freezing the aqueous solution in the wood leads to the expansion of its volume [83] leading to cracks. Specimens preserved with PEG exhibit low strength [18]. This fragility is further increased by the cracks that have formed as a result of the conservation.

#### Polyethylene glycol (PEG 400 and 4000) two-step and freeze-drying 

**Fig. 15:**
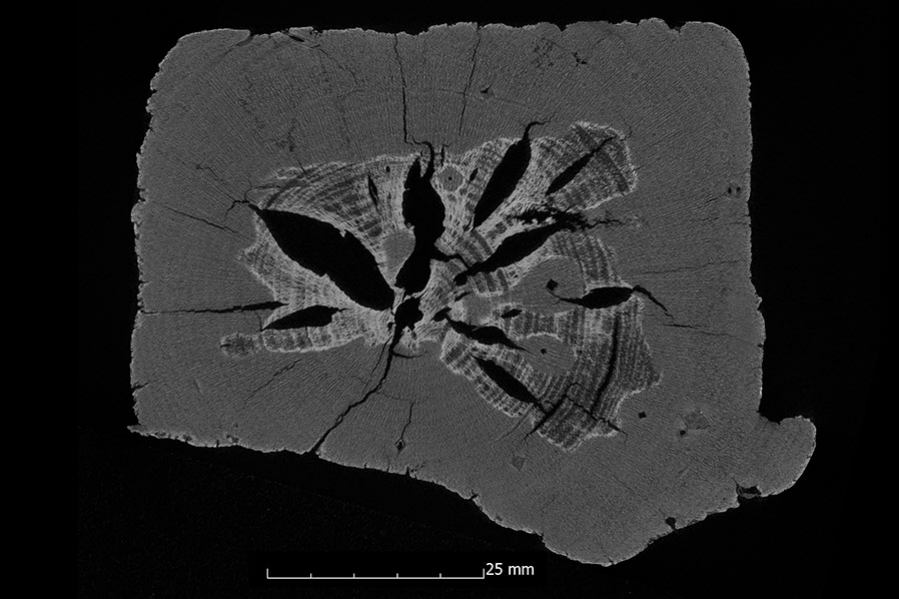
µCT cross section of the ash sample treated with PEG 400 and 4000 and freeze dried (As1-PEG2) with cracks in, and collapse of, the wood structure

The samples conserved in stages with PEG 400 and PEG 4000 and afterwards freeze-dried also show a predominantly favourable volume stabilisation (Appendix B). With the exception of the values of three samples, the calculated ASE is above 90%. The samples with the poorest values are the oak sample (Oa2-PEG2) with a value of 89%, the pine sample (Pi1-PEG2) with a value of 87%, and the second oak sample (Oa1-PEG2), which dropped sharply to 74%. The scans after 10 years show satisfactory volume stability, in line with the other conservation methods. Considering the results of the µCT, a similar picture appears as for the conservation with PEG 2000. The volume stabilisation inside the samples is good and with a value above 3%, the oak sample (Oa3-PEG2) has the highest additional shrinkage (Appendix C). An increase of the surface area with a good volume stabilisation inside the samples can also be observed, which in turn suggests the formation of cracks inside the samples (Appendix D). The cracks occur less strongly than with the other PEG methods. However, they are clearly visible in seven sample series. The fir samples (Fi1-PEG2, Fi2-PEG2) and the spruce sample (Sp1-PEG2) have no obvious cracks. In one case, in addition to cracks in the outer area, it can also be observed that the wood structure in the centre has collapsed (Fig. 15).

#### Polyethylene glycol (PEG 400, 1500 and 4000) three-step and freeze-drying

**Fig. 16:**
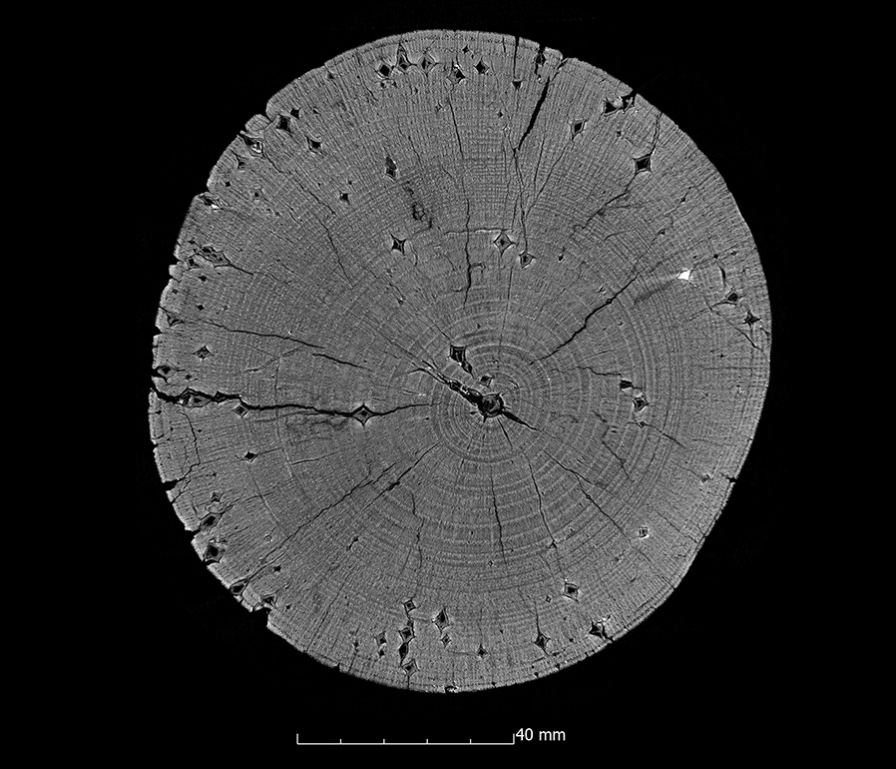
µCT cross section of the ash sample treated with PEG (three-step) and freeze dried (As1-PEG3) with cracks in the wood structure

**Fig. 17:**
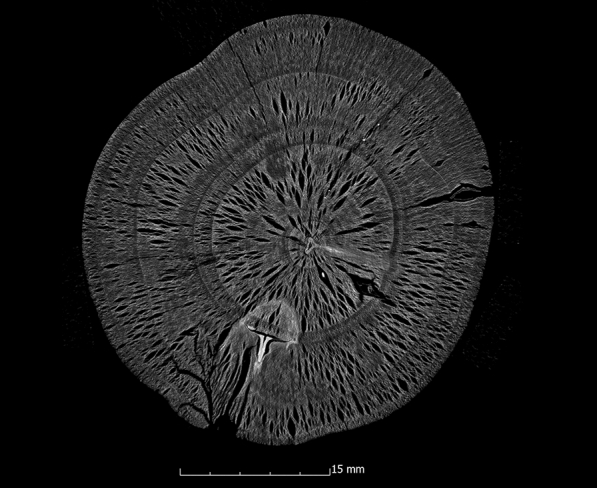
µCT cross section of the alder sample treated with PEG (three-step) and freeze dried (Al1-PEG3) with cracks in, and collapse of, the wood structure

The samples conserved in three stages with PEG 400, PEG 1500 and PEG 4000 and then freeze-dried are among the best results of the methods with PEG (Appendix B). With the exception of two samples, the ASE values are above 90%. The samples with poorer values are the oak sample (Oa1-PEG3) with a value of 85% and the pine sample (Pi1-PEG3) with a value of 80%. The scans after 10 years show satisfactory volume stability (Appendix C). As with the other conservation methods using PEG, the µCT data show good volume stabilisation with a simultaneous increase in surface area inside the samples, again indicating the formation of fine cracks (Appendix D). The µCT cross-sections (Fig. 16) confirm that these cracks occur in all samples conserved with the three-step PEG method except the fir sample (Fi1-PEG3). In particular, the alder sample (Al1-PEG3) stands out with a shrinkage of almost 7% and a surface area increase of over 600%. This sample is an exception among the samples conserved with PEG because, in addition to the cracks, there is also considerable collapse in the conserved wood structure (Fig. 17).

#### Saccharose

**Fig. 18:**
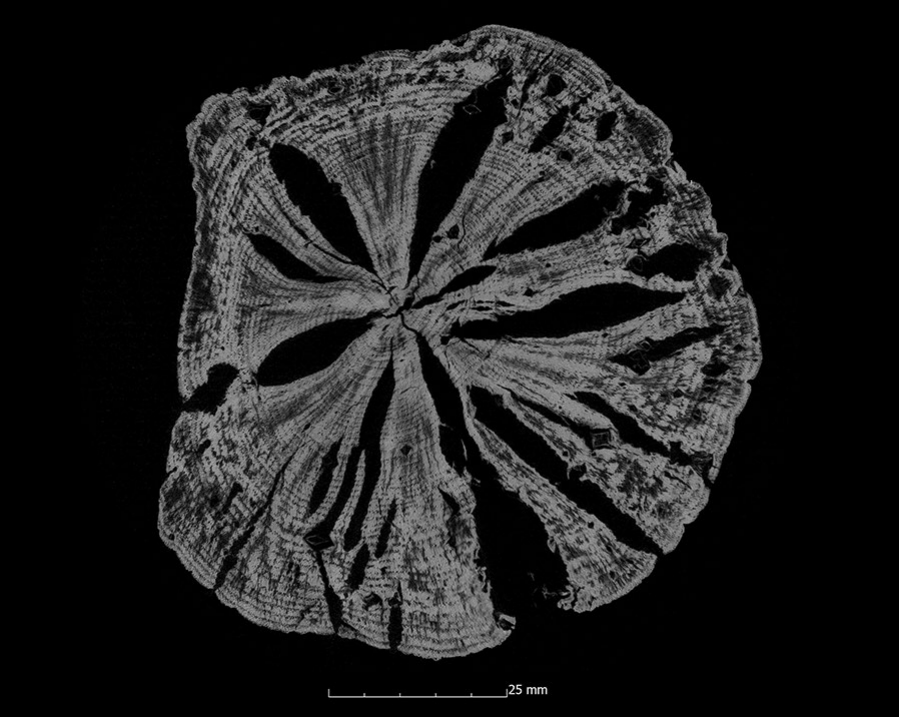
µCT cross section of the ash sample treated with saccharose (As1-Sac) with collapse of the wood structure

The samples conserved with saccharose show a poorer conservation overall and the results vary greatly. Thus, the ASE of the ten samples directly after conservation ranges between 64% and 94%, with only the fir sample (Fi1-Sac) with 94% and the pine sample (Pi1-Sac) with 90% showing a value above 90% (Appendix B). The ASE of the pine sample (Pi1-Sac) is particularly remarkable in this context, as the other conservation methods consistently perform rather poorly in this series of samples. A change in volume after ten years is also not observed in the wood samples conserved with saccharose (Fig. 7). The µCT data also show very different results, with very poor values of volume stabilisation inside the samples in some cases (Al1-Sac, As1-Sac, Oa3-Sac, Appendix C). Together with the high values of surface change (Appendix D), this indicates a significant collapse of the wood structure, as confirmed by the images of the ash sample (As1-Sac, Fig. 18). However, low stabilisation is not solely related to low concentration of consolidant. Highly concentrated solutions can also be problematic. Their susceptibility to degradation by osmotolerant microorganisms can lead to the development of slime and gases, which can prevent consolidants from penetrating the wood. Even an excess of biocides, due to the low penetration of the same, could not prevent these fermentation processes. The decomposition of saccharose takes place by chemical or microbiological processes, whereby the sugar is converted into the monosaccharides of fructose and glucose. This prevents volume stabilisation and, as the degradation products are more hygroscopic, leads to a damp surface and poorer drying [84–86].

#### Silicone oil

**Fig. 19:**
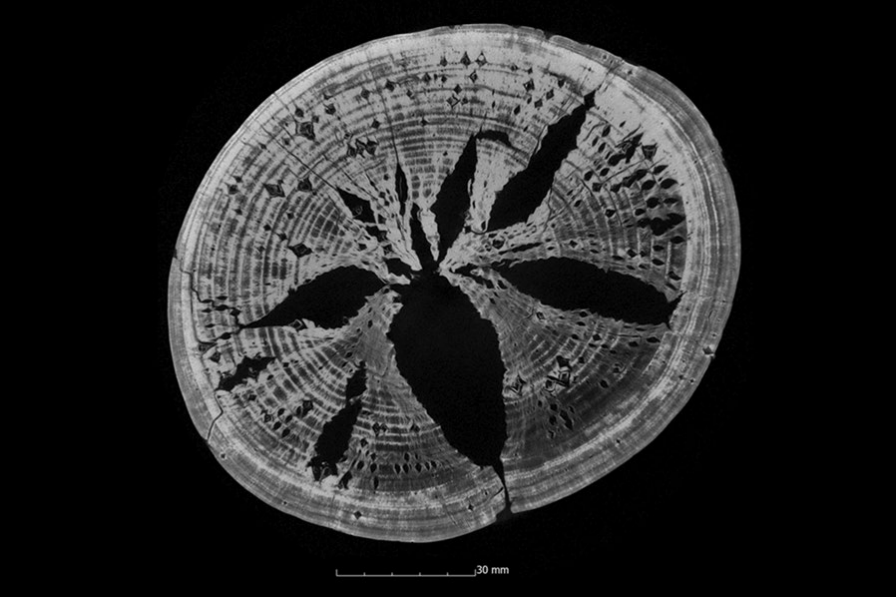
µCT cross section of the ash sample treated with silicone oil (As1-Sil) with collapse of the wood structure

The samples conserved with silicone oil tend to show poorer volume stabilisation overall and the ASE of the samples is between 71% and 89% (Appendix B). No change in volume can be observed in the wood conserved with silicone oil after ten years (Fig. 7). As with the saccharose-conserved samples, the µCT data show very different results (Appendix C). Thus, there are also striking samples (Al1-Sil, As1-Sil, Sp1-Sil) with poor volume stabilisation and an interior surface enlargement, which again indicates the collapse of the wood structure (Appendix D). This is also confirmed, for example, by the images of sample As1-Sil (Fig. 19).

## Conclusion

On the basis of the surface data from structured-light 3D scanning of ten test series with a total of 83 wood samples, it was possible to comparatively investigate the preservation results of different conservation methods with regard to their volume stabilisation. Wood is a very heterogeneous material and the successful conservation and volume stabilisation depends on different factors. This study showed that first of all the state of preservation is of great importance. The dimensional changes upon air-drying are dependent on the water content. This was also confirmed by other studies [2, 5, 13, 80].

There are also enormous differences between the conservation methods studied. It should be noted that all methods investigated in the study stabilised the wood to some extent, in contrast to air-drying. The investigation of the volume stability after 10 years further showed that all conserved samples remained stable in contrast to the air-dried samples. However, it should also be noted that only the methods using PEG, alcohol-ether-resin and Kauramin 800^®^ guarantee reliable volume stabilisation. The other methods were subject to considerable fluctuations and in some cases showed a large volume loss due to cell collapse.

For the first time, the internal structures of a large sample series were considered by the application of µCT. In contrast to the structured-light 3D scans performed here, the µCT measurements show the considerable damage such objects can take during conservation due to cracks and cell collapse. Such disadvantages could also be observed in samples that were treated with the methods using PEG, alcohol-ether-resin and Kauramin 800^®^. For example, in a condition with two differently degraded areas in the wood, large gaps can form during conservation with Kauramin 800^®^. This is possibly due to the fact that the conservation agent has the ability to stabilise heavily degraded wood better than well preserved wood, which may be explained by poorer penetration into well preserved wood [17, 87]. In the samples conserved with PEG and subsequently freeze-dried, a large number of fine cracks in the wood were very often visible in the µCT data. This can be explained by the fact that the aqueous PEG solution expands during freezing, resulting in damage to the wood structure. The two-step method using aqueous solutions of PEG 400 and PEG 4000 showed slightly better results with fewer cracks. This may be attributed to the fact that low molecular weight PEG lowers the critical freeze-drying temperature and a proportion of the solution does not freeze. Though less volume expansion may occur [46]. In addition, it was observed in one sample that there is considerable collapse in the centre. That may be explained by the fact that the aqueous solution of PEG thawed during the freeze-drying process. Afterwards, the wood structure collapsed when drying from the liquid phase. It is likely the conservation agent was transported to the outside areas by capillary forces, thus, appearing as though there is no conservation agent present in this area in the µCT data. This has already been described in the literature as being a crucial parameter in the conservation treatment with PEG [17, 18, 54, 58].

These observations show the importance of tomographic methods and the need to take into account the internal structures when assessing the conservation result. However, these observations also show that further research and validation of the results is required. In order to be able to assess the changes in the wood caused by the conservation with certainty, it is necessary in further test series to also record the preliminary condition of the samples using tomographic methods. However, in the case of the waterlogged condition of wood, it should be noted that the presence of water in the wood reduces the quality of the µCT data and complicates the measurement procedure. Therefore, for waterlogged wood, magnetic resonance imaging, for example, would be a suitable method to better capture the preliminary wet condition [55, 88, 89]. It should be mentioned that in this study only volume stabilisation of conservation methods was considered for evaluation. Other factors such as reversibility, effort, toxicity of the ingredients and long-term stability are also of great importance in the choice of the procedure.

## Data Availability

The datasets generated and/or analysed during the current study are available in the repository of the RGZM, [www.rgzm.de/kur]. Further datasets used and/or analysed during the current study are available from the corresponding author on reasonable request.

## Appendices

### Appendix A: Genus, conservation method, condition, dimensions and resolution µCT of the samples

**Table:**

Sample	KUR-No	Genus	Conservation method	Umax (%)	Condition (de Jong)	Basic density (g/cm^3^)	Residual basic density (%)	Dimensions (cm) L, Ø/L, W, H	Resolution µCT (µm)
Al1-Air	V24-23	Alder	Air dried	743	1	0.124	28	20, 3–5	27
Al1-AlEt	V24-45	Alder	Alcohol-Ether	706	1	0.129	29	12, 3–5	27
Al1-K800	V24-60	Alder	Kauramin 800^®^	958	1	0.098	22	16, 3–5	30
Al1-LaTr	V24-19	Alder	Lactitol/trehalose	891	1	0.104	24	14, 3–5	30
Al1-PEG1	V24-13	Alder	PEG (1)	853	1	0.109	25	17, 3–5	35
Al1-PEG2	V24-63	Alder	PEG (2)	671	1	0.136	31	18, 3–5	27
Al1-PEG3	V24-21	Alder	PEG (3)	1048	1	0.090	20	19, 3–5	30
Al1-Sac	V24-34	Alder	Saccharose	820	1	0.113	26	13, 3–5	27
Al1-Sil	V24-56	Alder	Silicone oil	807	1	0.114	26	15, 3–5	30
As1-Air	V28-27	Ash	Air dried	738	1	0.124	22	11, 12	43
As1-AlEt	V28-23	Ash	Alcohol-Ether	994	1	0.094	17	11, 12	44
As1-K800	V28-30	Ash	Kauramin 800^®^	961	1	0.097	17	11, 12	35
As1-PEG1	V28-08	Ash	PEG (1)	1291	1	0.074	13	11, 12	35
As1-PEG2	V28-10	Ash	PEG (2)	1195	1	0.079	14	11, 12	35
As1-PEG3	V28-36	Ash	PEG (3)	929	1	0.100	18	11, 12	44
As1-Sac	V28-07	Ash	Saccharose	1376	1	0.069	12	11, 12	35
As1-Sil	V28-24	Ash	Silicone oil	983	1	0.095	17	11, 12	35
Be1-Air	V07-23	Beech	Air dried	664	1	0.137	24	20, 8, 8	35
Be1-AlEt	V07-18	Beech	Alcohol-Ether	735	1	0.125	22	12, 8, 8	35
Be1-K800	V07-14	Beech	Kauramin 800^®^	720	1	0.127	23	19, 8, 8	35
Be1-LaTr	V07-Exp3	Beech	Lactitol/trehalose	566	1	0.158	28	17, 8, 8	35
Be1-PEG1	V07-09	Beech	PEG (1)	772	1	0.119	21	13, 8, 8	35
Be1-PEG2	V07-27	Beech	PEG (2)	654	1	0.139	25	14, 8, 8	35
Be1-PEG3	V07-08	Beech	PEG (3)	747	1	0.123	22	15, 8, 8	35
Be1-Sac	V07-01	Beech	Saccharose	748	1	0.123	22	16, 8, 8	35
Be1-Sil	V07-37	Beech	Silicone oil	808	1	0.114	20	18, 8, 8	35
Fi1-Air	V22-28	Fir	Air dried	381	2	0.223	59	30, 8, 2,5	32
Fi1-AlEt	V22-09	Fir	Alcohol-Ether	398	2	0.215	57	22, 8, 2,5	32
Fi1-K800	V22-33	Fir	Kauramin 800^®^	487	1	0.181	48	26, 8, 2,5	32
Fi1-LaTr	V22-26	Fir	Lactitol/trehalose	352	2	0.239	63	24, 8, 2,5	32
Fi1-PEG1	V22-22	Fir	PEG (1)	432	1	0.201	53	27, 8, 2,5	32
Fi1-PEG2	V22-31	Fir	PEG (2)	371^1^	2	0.228^1^	60^1^	28, 8, 2,5	32
Fi1-PEG3	V22-35	Fir	PEG (3)	241	2	0.325	86	29, 8, 2,5	32
Fi1-Sac	V22-11	Fir	Saccharose	285	2	0.284	75	23, 8, 2,5	32
Fi1-Sil	V22-05	Fir	Silicone oil	333	2	0.250	66	25, 8, 2,5	32
Fi2-Air	V30-Exp1	Fir	Air dried	465	1	0.188	49	11, 6–7	40
Fi2-AlEt	V30-33	Fir	Alcohol-Ether	695	1	0.131	35	11, 6–7	40
Fi2-K800	V30-09	Fir	Kauramin 800^®^	601	1	0.150	39	11, 6–7	40
Fi2-PEG1	V30-04	Fir	PEG (1)	542	1	0.164	43	11, 6–7	40
Fi2-PEG2	V30-18	Fir	PEG (2)	632	1	0.143	38	11, 6–7	40
Fi2-PEG3	V30-15	Fir	PEG (3)	554	1	0.161	42	11, 6–7	40
Fi2-Sac	V30-17	Fir	Saccharose	599	1	0.150	40	11, 6–7	40
Fi2-Sil	V30-24	Fir	Silicone oil	548	1	0.163	43	11, 6–7	40
Oa1-Air	V10-02	Oak	Air dried	201	2	0.374	67	17, 7	32
Oa1-K800	V10-06	Oak	Kauramin 800^®^	198	2	0.378	67	16, 7	32
Oa1-LaTr	V10-33	Oak	Lactitol/trehalose	160	3	0.441	79	15, 7	32
Oa1-PEG1	V10-29	Oak	PEG (1)	179	3	0.407	73	11, 7	32
Oa1-PEG2	V10-31	Oak	PEG (2)	176	3	0.412	74	12, 7	32
Oa1-PEG3	V10-39	Oak	PEG (3)	181	3	0.404	72	13, 7	32
Oa1-Sac	V10-03	Oak	Saccharose	163	3	0.435	78	14, 7	32
Oa2-Air	V16-15	Oak	Air dried	362	2	0.233	42	12, 15, 4,5	33
Oa2-AlEt	V16-09	Oak	Alcohol-Ether	175	3	0.414	74	12, 15, 4,5	32
Oa2-K800	V16-25	Oak	Kauramin 800^®^	228	2	0.339	61	12, 15, 4,5	44
Oa2-PEG1	V16-18	Oak	PEG (1)	189	2	0.391	70	12, 15, 4,5	35
Oa2-PEG2	V16-32	Oak	PEG (2)	237	2	0.329	59	12, 15, 4,5	44
Oa2-PEG3	V16-36	Oak	PEG (3)	188	2	0.393	70	12, 15, 4,5	35
Oa2-Sac	V16-17	Oak	Saccharose	184	3	0.399	71	12, 15, 4,5	35
Oa2-Sil	V16-13	Oak	Silicone oil	187	2	0.394	70	12, 15, 4,5	32
Oa3-Air	V27-17	Oak	Air dried	577	1	0.155	28	12–14, 5–6	19
Oa3-AlEt	V27-40	Oak	Alcohol-ether	527	1	0.168	30	12–14, 5–6	32
Oa3-K800	V27-07	Oak	Kauramin 800^®^	646	1	0.140	25	12–14, 5–6	26
Oa3-LaTr	V27-30	Oak	Lactitol/trehalose	566	1	0.158	28	12–14, 5–6	26
Oa3-PEG1	V27-12	Oak	PEG (1)	691	1	0.132	24	12–14, 5–6	32
Oa3-PEG2	V27-02	Oak	PEG (2)	578	1	0.155	28	12–14, 5–6	32
Oa3-PEG3	V27-01	Oak	PEG (3)	557	1	0.160	29	12–14, 5–6	39
Oa3-Sac	V27-21	Oak	Saccharose	434	1	0.200	36	12–14, 5–6	26
Oa3-Sil	V27-10	Oak	Silicone oil	520	1	0.170	30	12–14, 5–6	26
Pi1-Air	V03-01	Pine	Air dried	390	2	0.219	52	20, 7, 9–12	39
Pi1-AlEt	V03-17	Pine	Alcohol-Ether	332^1^	2	0.251^1^	60^1^	12, 7, 9–12	40
Pi1-K800	V03-41	Pine	Kauramin 800^®^	332^1^	2	0.251^1^	60^1^	19, 7, 9–12	40
Pi1-LaTr	V03-28	Pine	Lactitol/trehalose	332^1^	2	0.251^1^	60^1^	17, 7, 9–12	40
Pi1-PEG1	V03-35	Pine	PEG (1)	332^1^	2	0.251^1^	60^1^	13, 7, 9–12	40
Pi1-PEG2	V03-32	Pine	PEG (2)	332^1^	2	0.251^1^	60^1^	14, 7, 9–12	40
Pi1-PEG3	V03-20	Pine	PEG (3)	332^1^	2	0.251^1^	60^1^	15, 7, 9–12	40
Pi1-Sac	V03-45	Pine	Saccharose	332^1^	2	0.251^1^	60^1^	16, 7, 9–12	40
Pi1-Sil	V03-42	Pine	Silicone oil	332^1^	2	0.251^1^	60^1^	18, 7, 9–12	40
Sp1-Air	V23-20	Spruce	Air dried	503	1	0.176	46	21, 10, 6	40
Sp1-K800	V23-08	Spruce	Kauramin 800^®^	482	1	0.182	48	20, 10, 6	40
Sp1-LaTr	V23-02	Spruce	Lactitol/trehalose	454	1	0.192	51	19, 10, 6	40
Sp1-PEG1	V23-25	Spruce	PEG (1)	427	1	0.203	53	15, 10, 6	40
Sp1-PEG2	V23-30	Spruce	PEG (2)	280	2	0.288	76	16, 10, 6	40
Sp1-PEG3	V23-28	Spruce	PEG (3)	282	2	0.287	75	17, 10, 6	40
Sp1-Sac	V23-29	Spruce	Saccharose	374	2	0.227	60	18, 10, 6	40

1 = Mean value of the sample series

### Appendix B: Volumes of the samples before and after conservation with calculated shrinkage and ASE

**Table:**

Sample	KUR-No	Volume Scan1 (mm^3^) before conservation	Volume Scan2 (mm^3^) after conservation	Shrinkage (%)	ASE (%)
Al1-Air	V24-23	146,308	31,179	78.69	**0**
Al1-AlEt	V24-45	102,902	99,115	3.68	**95**
Al1-K800	V24-60	160,907	162,261	− 0.84	**101**
Al1-LaTr	V24-19	128,825	104,028	19.25	**76**
Al1-PEG1	V24-13	224,601	223,555	0.47	**99**
Al1-PEG2	V24-63	105,005	104,819	0.18	**95**
Al1-PEG3	V24-21	159,932	156,425	2.19	**97**
Al1-Sac	V24-34	113,119	81,711	27.77	**65**
Al1-Sil	V24-56	154,675	140,245	9.33	**88**
As1-Air	V28-27	1,333,063	235,173	82.36	**0**
As1-AlEt	V28-23	1,243,400	1,205,707	3.03	**96**
As1-K800	V28-30	571,461	573,401	− 0.34	**100**
As1-PEG1	V28-08	731,204	755,998	− 3.39	**104**
As1-PEG2	V28-10	416,108	421,655	− 1.33	**102**
As1-PEG3	V28-36	1,121,247	1,145,266	− 2.14	**103**
As1-Sac	V28-07	757,823	604,063	20.29	**75**
As1-Sil	V28-24	1,220,413	1,099,338	9.92	**88**
Be1-Air	V07-23	772,702	151,476	80.40	**0**
Be1-AlEt	V07-18	597,620	565,700	5.34	**93**
Be1-K800	V07-14	689,166	688,499	0.10	**100**
Be1-LaTr	V07-Exp3	671,849	630,914	6.09	**92**
Be1-PEG1	V07-09	674,679	671,609	0.46	**99**
Be1-PEG2	V07-27	699,016	697,988	0.15	**100**
Be1-PEG3	V07-08	585,750	569,672	2.74	**97**
Be1-Sac	V07-01	609,858	434,048	28.83	**64**
Be1-Sil	V07-37	507,941	389,231	23.37	**71**
Fi1-Air	V22-28	453,549	222,550	50.93	**0**
Fi1-AlEt	V22-09	460,970	440,617	4.42	**91**
Fi1-K800	V22-33	384,611	371,581	3.39	**93**
Fi1-LaTr	V22-26	360,264	328,850	8.72	**83**
Fi1-PEG1	V22-22	494,810	478,138	3.37	**93**
Fi1-PEG2	V22-31	445,544	434,733	2.43	**95**
Fi1-PEG3	V22-35	500,516	483,497	3.40	**93**
Fi1-Sac	V22-11	480,933	465,727	3.16	**94**
Fi1-Sil	V22-05	434,348	392,397	9.66	**81**
Fi2-Air	V30-Exp1	88,000	49,539	43.71	**0**
Fi2-AlEt	V30-33	427,312	414,753	2.94	**93**
Fi2-K800	V30-09	495,437	492,590	0.57	**99**
Fi2-PEG1	V30-04	307,024	306,696	0.11	**100**
Fi2-PEG2	V30-18	269,965	270,071	− 0.04	**100**
Fi2-PEG3	V30-15	421,682	411,711	2.36	**95**
Fi2-Sac	V30-17	357,884	338,088	5.53	**87**
Fi2-Sil	V30-24	404,852	386,070	4.64	**89**
Oa1-Air	V10-02	725,355	399,675	44.90	**0**
Oa1-K800	V10-06	895,292	868,531	2.99	**93**
Oa1-LaTr	V10-33	768,421	705,640	8.17	**82**
Oa1-PEG1	V10-29	1,008,166	930,839	7.67	**83**
Oa1-PEG2	V10-31	997,903	883,538	11.46	**74**
Oa1-PEG3	V10-39	969,533	883,916	8.83	**80**
Oa1-Sac	V10-03	901,297	798,944	11.36	**75**
Oa2-Air	V16-15	512,214	249,991	51.19	**0**
Oa2-AlEt	V16-09	806,657	758,211	6.01	**88**
Oa2-K800	V16-25	1,214,955	1,160,158	4.51	**91**
Oa2-PEG1	V16-18	1,002,133	944,715	5.73	**89**
Oa2-PEG2	V16-32	1,258,876	1,188,430	5.60	**89**
Oa2-PEG3	V16-36	1,208,876	1,157,459	4.25	**92**
Oa2-Sac	V16-17	1,056,104	961,673	8.94	**83**
Oa2-Sil	V16-13	844,580	725,961	14.04	**73**
Oa3-Air	V27-17	293,261	47,476	83.81	**0**
Oa3-AlEt	V27-40	267,820	250,129	6.61	**92**
Oa3-K800	V27-07	340,889	338,726	0.63	**99**
Oa3-LaTr	V27-30	266,238	220,194	17.29	**79**
Oa3-PEG1	V27-12	363,884	362,077	0.50	**99**
Oa3-PEG2	V27-02	283,059	268,144	5.27	**94**
Oa3-PEG3	V27-01	289,788	266,534	8.02	**90**
Oa3-Sac	V27-21	316,392	249,489	21.15	**75**
Oa3-Sil	V27-10	379,014	325,680	14.07	**83**
Pi1-Air	V03-01	901,457	693,464	23.07	**0**
Pi1-AlEt	V03-17	771,098	727,558	5.65	**76**
Pi1-K800	V03-41	1,067,031	1,034,388	3.06	**87**
Pi1-LaTr	V03-28	776,356	755,903	2.63	**89**
Pi1-PEG1	V03-35	867,035	849,403	2.03	**91**
Pi1-PEG2	V03-32	983,539	953,583	3.05	**87**
Pi1-PEG3	V03-20	747,056	721,864	3.37	**85**
Pi1-Sac	V03-45	752,562	734,681	2.38	**90**
Pi1-Sil	V03-42	783,823	739,365	5.67	**75**
Sp1-Air	V23-20	1,062,100	365,165	65.62	**0**
Sp1-K800	V23-08	906,595	850,773	6.16	**91**
Sp1-LaTr	V23-02	839,765	768,510	8.49	**87**
Sp1-PEG1	V23-25	1,031,354	1,063,448	− 3.11	**105**
Sp1-PEG2	V23-30	955,998	944,487	1.20	**98**
Sp1-PEG3	V23-28	1,053,538	1,047,572	0.57	**99**
Sp1-Sac	V23-29	885,828	771,872	12.86	**80**

### Appendix C: Volume changes of the samples 10 years after conservation considering inner structures from µCT

**Table:**

Sample	KUR-No	Volume Scan2 (mm^3^) after conservation	Volume Scan3 (mm^3^) 10 years after conservation	Volume µCT (mm^3^) 10 years after conservation	Shrinkage (%) Scan 2 and 3	Shrinkage (%) Scan 3 and µCT
Al1-Air	V24-23	31,179	24,760	17,706	**20.59**	**28.49**
Al1-AlEt	V24-45	99,115	98,384	93,231	**0.74**	**5.24**
Al1-K800	V24-60	162,261	161,185	156,812	**0.66**	**2.71**
Al1-LaTr	V24-19	104,028	102,996	86,192	**0.99**	**16.31**
Al1-PEG1	V24-13	223,555	223,018	217,914	**0.24**	**2.29**
Al1-PEG2	V24-63	104,819	102,585	100,481	**2.13**	**2.05**
Al1-PEG3	V24-21	156,425	154,216	143,465	**1.41**	**6.97**
Al1-Sac	V24-34	81,711	78,017	68,037	**4.52**	**12.79**
Al1-Sil	V24-56	140,245	139,209	132,425	**0.74**	**4.87**
As1-Air	V28-27	235,173	216,694	193,375	**7.86**	**10.76**
As1-AlEt	V28-23	1,205,707	1,197,414	1,184,689	**0.69**	**1.06**
As1-K800	V28-30	573,401	569,276	564,613	**0.72**	**0.82**
As1-PEG1	V28-08	755,998	754,140	744,974	**0.25**	**1.22**
As1-PEG2	V28-10	421,655	419,105	410,026	**0.60**	**2.17**
As1-PEG3	V28-36	1,145,266	1,147,825	1,137,275	**− 0.22**	**0.92**
As1-Sac	V28-07	604,063	597,524	512,904	**1.08**	**14.16**
As1-Sil	V28-24	1,099,338	1,089,794	939,898	**0.87**	**13.75**
Be1-Air	V07-23	151,476	147,738	127,144	**2.47**	**13.94**
Be1-AlEt	V07-18	565,700	559,303	558,498	**1.13**	**0.14**
Be1-K800	V07-14	688,499	686,595	677,402	**0.28**	**1.34**
Be1-LaTr	V07-Exp3	630,914	630,508	593,651	**0.06**	**5.85**
Be1-PEG1	V07-09	671,609	668,946	662,300	**0.40**	**0.99**
Be1-PEG2	V07-27	697,988	693,637	681,991	**0.62**	**1.68**
Be1-PEG3	V07-08	569,672	566,796	559,662	**0.50**	**1.26**
Be1-Sac	V07-01	434,048	433,702	427,680	**0.08**	**1.39**
Be1-Sil	V07-37	389,231	390,118	379,094	**-0.23**	**2.83**
Fi1-Air	V22-28	222,550	219,359	215,214	**1.43**	**1.89**
Fi1-AlEt	V22-09	440,617	437,768	434,591	**0.65**	**0.73**
Fi1-K800	V22-33	371,581	369,032	364,899	**0.69**	**1.12**
Fi1-LaTr	V22-26	328,850	326,867	326,097	**0.60**	**0.24**
Fi1-PEG1	V22-22	478,138	478,878	478,834	**− 0.15**	**0.01**
Fi1-PEG2	V22-31	434,733	436,025	433,782	**− 0.30**	**0.51**
Fi1-PEG3	V22-35	483,497	483,900	483,138	**− 0.08**	**0.16**
Fi1-Sac	V22-11	465,727	464,262	461,471	**0.31**	**0.60**
Fi1-Sil	V22-05	392,397	393,697	389,657	**− 0.33**	**1.03**
Fi2-Air	V30-Exp1	49,539	48,737	45,156	**1.62**	**7.35**
Fi2-AlEt	V30-33	414,753	413,424	409,599	**0.32**	**0.93**
Fi2-K800	V30-09	492,590	491,311	486,574	**0.26**	**0.96**
Fi2-PEG1	V30-04	306,696	305,457	298,768	**0.40**	**2.19**
Fi2-PEG2	V30-18	270,071	269,760	265,858	**0.12**	**1.45**
Fi2-PEG3	V30-15	411,711	412,560	403,988	**− 0.21**	**2.08**
Fi2-Sac	V30-17	338,088	336,763	330,397	**0.39**	**1.89**
Fi2-Sil	V30-24	386,070	385,594	384,166	**0.12**	**0.37**
Oa1-Air	V10-02	399,675	375,634	360,929	**6.02**	**3.91**
Oa1-K800	V10-06	868,531	852,548	760,510	**1.84**	**10.80**
Oa1-LaTr	V10-33	705,640	684,208	679,196	**3.04**	**0.73**
Oa1-PEG1	V10-29	930,839	925,324	922,177	**0.59**	**0.34**
Oa1-PEG2	V10-31	883,538	873,966	871,588	**1.08**	**0.27**
Oa1-PEG3	V10-39	883,916	884,323	863,624	**− 0.05**	**2.34**
Oa1-Sac	V10-03	798,944	800,842	798,595	**− 0.24**	**0.28**
Oa2-Air	V16-15	249,991	242,620	232,541	**2.95**	**4.15**
Oa2-AlEt	V16-09	758,211	746,747	746,471	**1.51**	**0.04**
Oa2-K800	V16-25	1,160,158	1,159,215	980,995	**0.08**	**15.37**
Oa2-PEG1	V16-18	944,715	944,951	927,353	**− 0.02**	**1.86**
Oa2-PEG2	V16-32	1,188,430	1,175,774	1,171,048	**1.06**	**0.40**
Oa2-PEG3	V16-36	1,157,459	1,148,261	1,131,193	**0.79**	**1.49**
Oa2-Sac	V16-17	961,673	965,032	960,526	**− 0.35**	**0.47**
Oa2-Sil	V16-13	725,961	723,683	722,258	**0.31**	**0.20**
Oa3-Air	V27-17	47,476	44,990	41,674	**5.24**	**7.37**
Oa3-AlEt	V27-40	250,129	245,888	243,032	**1.70**	**1.16**
Oa3-K800	V27-07	338,726	338,272	325,887	**0.13**	**3.66**
Oa3-LaTr	V27-30	220,194	220,472	189,715	**− 0.13**	**13.95**
Oa3-PEG1	V27-12	362,077	360,424	356,083	**0.46**	**1.20**
Oa3-PEG2	V27-02	268,144	266,536	258,136	**0.60**	**3.15**
Oa3-PEG3	V27-01	266,534	269,946	261,177	**− 1.28**	**3.25**
Oa3-Sac	V27-21	249,489	246,670	226,558	**1.13**	**8.15**
Oa3-Sil	V27-10	325,680	318,458	311,940	**2.22**	**2.05**
Pi1-Air	V03-01	693,464	690,464	669,462	**0.43**	**3.04**
Pi1-AlEt	V03-17	727,558	720,240	715,479	**1.01**	**0.66**
Pi1-K800	V03-41	1,034,388	1,029,360	1,024,444	**0.49**	**0.48**
Pi1-LaTr	V03-28	755,903	747,161	744,845	**1.16**	**0.31**
Pi1-PEG1	V03-35	849,403	848,117	844,877	**0.15**	**0.38**
Pi1-PEG2	V03-32	953,583	941,285	937,230	**1.29**	**0.43**
Pi1-PEG3	V03-20	721,864	723,021	720,784	**− 0.16**	**0.31**
Pi1-Sac	V03-45	734,681	736,946	733,326	**− 0.31**	**0.49**
Pi1-Sil	V03-42	739,365	745,165	738,476	**− 0.78**	**0.90**
Sp1-Air	V23-20	365,165	355,505	350,248	**2.65**	**1.48**
Sp1-K800	V23-08	850,773	842,600	821,603	**0.96**	**2.49**
Sp1-LaTr	V23-02	768,510	759,002	728,992	**1.24**	**3.95**
Sp1-PEG1	V23-25	1,063,448	1,059,582	1,053,036	**0.36**	**0.62**
Sp1-PEG2	V23-30	944,487	937,396	933,264	**0.75**	**0.44**
Sp1-PEG3	V23-28	1,047,572	1,048,511	1,045,822	**− 0.09**	**0.26**
Sp1-Sac	V23-29	771,872	766,530	745,213	**0.69**	**2.78**

### Appendix D: Surface and volume changes of the samples 10 years after conservation considering inner structures from µCT

**Table:**

Sample	KUR-No	Surface Scan3 (mm^2^) 10 years after conservation	Surface µCT (mm^2^) 10 years after conservation	Surface change (%) Scan 3 and µCT	Shrinkage (%) Scan 3 and µCT
Al1-Air	V24-23	12,415	35,734	**188**	**28.49**
Al1-AlEt	V24-45	14,420	34,211	**137**	**5.24**
Al1-K800	V24-60	20,390	49,633	**143**	**2.71**
Al1-LaTr	V24-19	21,314	86,288	**305**	**16.31**
Al1-PEG1	V24-13	23,221	80,711	**248**	**2.29**
Al1-PEG2	V24-63	14,962	30,272	**102**	**2.05**
Al1-PEG3	V24-21	18,914	140,336	**642**	**6.97**
Al1-Sac	V24-34	16,940	37,182	**119**	**12.79**
Al1-Sil	V24-56	18,652	38,461	**106**	**4.87**
As1-Air	V28-27	35,082	119,044	**239**	**10.76**
As1-AlEt	V28-23	63,506	107,480	**69**	**1.06**
As1-K800	V28-30	44,077	87,917	**99**	**0.82**
As1-PEG1	V28-08	48,955	155,842	**218**	**1.22**
As1-PEG2	V28-10	37,882	96,622	**155**	**2.17**
As1-PEG3	V28-36	65,935	148,547	**125**	**0.92**
As1-Sac	V28-07	44,404	208,448	**369**	**14.16**
As1-Sil	V28-24	63,532	220,025	**246**	**13.75**
Be1-Air	V07-23	26,021	84,639	**225**	**13.94**
Be1-AlEt	V07-18	44,767	54,400	**22**	**0.14**
Be1-K800	V07-14	48,788	67,928	**39**	**1.34**
Be1-LaTr	V07-Exp3	48,653	168,337	**246**	**5.85**
Be1-PEG1	V07-09	48,109	119,721	**149**	**0.99**
Be1-PEG2	V07-27	49,745	132,591	**167**	**1.68**
Be1-PEG3	V07-08	43,384	73,579	**70**	**1.26**
Be1-Sac	V07-01	39,509	57,242	**45**	**1.39**
Be1-Sil	V07-37	37,812	70,962	**88**	**2.83**
Fi1-Air	V22-28	43,576	60,517	**39**	**1.89**
Fi1-AlEt	V22-09	53,868	66,645	**24**	**0.73**
Fi1-K800	V22-33	50,126	86,374	**72**	**1.12**
Fi1-LaTr	V22-26	50,801	67,624	**33**	**0.24**
Fi1-PEG1	V22-22	55,726	59,109	**6**	**0.01**
Fi1-PEG2	V22-31	56,394	78,984	**40**	**0.51**
Fi1-PEG3	V22-35	57,206	65,082	**12**	**0.16**
Fi1-Sac	V22-11	53,339	71,043	**33**	**0.60**
Fi1-Sil	V22-05	53,077	65,926	**24**	**1.03**
Fi2-Air	V30-Exp1	8635	19,131	**122**	**7.35**
Fi2-AlEt	V30-33	32,416	51,646	**59**	**0.93**
Fi2-K800	V30-09	37,876	86,145	**127**	**0.96**
Fi2-PEG1	V30-04	26,653	90,152	**238**	**2.19**
Fi2-PEG2	V30-18	26,381	71,852	**172**	**1.45**
Fi2-PEG3	V30-15	34,819	100,612	**189**	**2.08**
Fi2-Sac	V30-17	29,908	71,373	**139**	**1.89**
Fi2-Sil	V30-24	31,114	38,846	**25**	**0.37**
Oa1-Air	V10-02	52,255	85,432	**63**	**3.91**
Oa1-K800	V10-06	61,457	131,774	**114**	**10.80**
Oa1-LaTr	V10-33	57,718	102,110	**77**	**0.73**
Oa1-PEG1	V10-29	62,422	88,035	**41**	**0.34**
Oa1-PEG2	V10-31	58,833	81,532	**39**	**0.27**
Oa1-PEG3	V10-39	61,296	122,801	**100**	**2.34**
Oa1-Sac	V10-03	56,280	68,620	**22**	**0.28**
Oa2-Air	V16-15	35,080	59,160	**69**	**4.15**
Oa2-AlEt	V16-09	57,645	89,005	**54**	**0.04**
Oa2-K800	V16-25	81,202	198,276	**144**	**15.37**
Oa2-PEG1	V16-18	73,083	151,386	**107**	**1.86**
Oa2-PEG2	V16-32	80,898	134,853	**67**	**0.40**
Oa2-PEG3	V16-36	78,207	131,352	**68**	**1.49**
Oa2-Sac	V16-17	73,739	163,807	**122**	**0.47**
Oa2-Sil	V16-13	56,663	76,156	**34**	**0.20**
Oa3-Air	V27-17	11,681	23,883	**104**	**7.37**
Oa3-AlEt	V27-40	29,406	41,819	**42**	**1.16**
Oa3-K800	V27-07	31,023	62,587	**102**	**3.66**
Oa3-LaTr	V27-30	31,972	146,691	**359**	**13.95**
Oa3-PEG1	V27-12	31,420	49,818	**59**	**1.20**
Oa3-PEG2	V27-02	34,300	81,133	**137**	**3.15**
Oa3-PEG3	V27-01	32,071	62,450	**95**	**3.25**
Oa3-Sac	V27-21	37,746	101,695	**169**	**8.15**
Oa3-Sil	V27-10	36,179	47,156	**30**	**2.05**
Pi1-Air	V03-01	56,362	70,166	**24**	**3.04**
Pi1-AlEt	V03-17	52,635	71,872	**37**	**0.66**
Pi1-K800	V03-41	69,860	98,177	**41**	**0.48**
Pi1-LaTr	V03-28	58,601	63,425	**8**	**0.31**
Pi1-PEG1	V03-35	59,624	93,104	**56**	**0.38**
Pi1-PEG2	V03-32	65,219	94,048	**44**	**0.43**
Pi1-PEG3	V03-20	52,700	68,616	**30**	**0.31**
Pi1-Sac	V03-45	54,560	61,158	**12**	**0.49**
Pi1-Sil	V03-42	54,128	72,204	**33**	**0.90**
Sp1-Air	V23-20	45,100	98,004	**117**	**1.48**
Sp1-K800	V23-08	60,128	177,153	**195**	**2.49**
Sp1-LaTr	V23-02	57,273	206,642	**261**	**3.95**
Sp1-PEG1	V23-25	68,803	144,463	**110**	**0.62**
Sp1-PEG2	V23-30	68,719	95,985	**28**	**0.44**
Sp1-PEG3	V23-28	70,485	107,966	**35**	**0.26**
Sp1-Sac	V23-29	56,646	226,761	**300**	**2.78**

## References

[1] Nilsson T, Rowell R (2012). Historical wood—structure and properties. J Cult Herit.

[2] Hoffmann P (2013). Conservation of archaeological ships and boats: personal experiences.

[3] Fengel D, Wegener G (2003). Wood: chemistry, ultrastructure, reactions.

[4] Kim YS, Singh AP (2000). Micromorphological characteristics of wood biodegradation in wet environments: a review. IAWA J.

[5] Hoffmann P, Jones MA, Rowell RM, Barbour J (1990). Structure and degradation process for waterlogged archaeological wood. Archaeological wood; properties, chemistry and preservation.

[6] Blanchette RA (2000). A review of microbial deterioration found in archaeological wood from different environments. Int Biodeterior.

[7] Björdal CG (2012). Microbial degradation of waterlogged archaeological wood. J Cult Herit.

[8] Grattan DW, Pearson C (1987). Waterlogged wood. Conservation of marine archaeological objects.

[9] High KE, Penkman KEH (2020). A review of analytical methods for assessing preservation in waterlogged archaeological wood and their application in practice. Herit Sci.

[10] Schniewind AP, Rowell RM, Barbour J (1990). Physical and mechanical properties of archaeological wood. Archaeological wood; properties, chemistry and preservation.

[11] Barbour RJ, Leney L. Shrinkage and collapse in waterlogged archaeological wood: Contribution III, Hoko River Series. In: Grattan DW, McCawley JC. editors. Proceedings of the ICOM-CC waterlogged wood working group conference, Ottawa, 1981. Ottawa: ICOM-CC; 1982. p. 208–25.

[12] Hawley OF. Wood-liquid relations. Technical bulletin, no. 248. Washington: United States Department of Agriculture, 1931.

[13] Christensen BB, Kongelike Nordiske Oldskriftselskan (1952). Om Konservering af Mosefundne Trægenstande. Aarbøger for Nordisk Oldkyndighed og Historie 1951.

[14] Grattan DW, Clarke RW, Pearson C (1987). Conservation of waterlogged wood. Conservation of marine archaeological objects.

[15] Grattan DW, McCawley JC (1978). The potential of the canadian winter climate for the freeze-drying of degraded waterlogged wood. Stud Conserv.

[16] Grattan DW (1982). A practical comparative study of several treatments for waterlogged wood. Stud Conserv.

[17] Hoffmann P. On the efficiency of stabilisation methods for large waterlogged wooden objects, and on how to choose a method. In: Straetkver K, Huisman DJ, editors. Proceedings of the 10th ICOM-CC Group on wet organic archaeological materials conference, Amsterdam, 2007. Amersfoort: Rijksdienst Voor Archeologie, Cultuurlandschap En Monumenten; 2009. p. 323–50.

[18] Bräker OU, Bill J, Mühlethaler B, Schoch W, Schweingruber FH, Haas A (1979). Zum derzeitigen Stand der Nassholzkonservierung. Diskussion der Grundlagen und Resultate eines von Fachlaboratorien 1976–1978 durchgeführten Methodenvergleiches. Zeitschr f Schweiz Archaeol Kunstgesch.

[19] Christensen BB (1971). Developments in the treatment of waterlogged wood in the National Museum of Denmark during the years 1962–69. Stud Conserv.

[20] International Council of Museums (2017). ICOM code of ethics for museums.

[21] Florian M-LE, Rowell RM, Barbour J (1990). Scope and history of archaeological wood. Archaeological wood; properties, chemistry and preservation.

[22] Jenssen V, Pearson C (1987). Conservation of wet organic artefacts excluding wood. Conservation of marine archaeological objects.

[23] Herbst CF. Om bevaring af oldsager af træ fundne i törvemoser. Antiquarisk tidsskrift. Kjøbenhavn: Det kongelige nordiske oldskriftselskab; 1861. p. 174–6.

[24] Rathgen F (1924). Die Konservierung von Altertumsfunden, Teil 2/3: Metalle und Metallegierungen, organische Stoffe: Mit Berücksichtigung ethnographischer und kunstgewerblicher Sammlungsgegenstände.

[25] Organ RM (1959). Carbowax and other materials in the treatment of water-logged paleolithic wood. Stud Conserv.

[26] Parrent JM (1985). The conservation of waterlogged wood using sucrose. Stud Conserv.

[27] Rosenqvist AM (1959). The stabilizing of wood found in the Viking ship of Oseberg, Pt. II. Stud Conserv.

[28] Broda M, Dąbek I, Dutkiewicz A, Dutkiewicz M, Popescu C-M, Mazela B (2020). Organosilicons of different molecular size and chemical structure as consolidants for waterlogged archaeological wood—a new reversible and retreatable method. Sci Rep.

[29] Walsh Z, Janeček E-R, Hodgkinson JT, Sedlmair J, Koutsioubas A, Spring DR (2014). Multifunctional supramolecular polymer networks as next-generation consolidants for archaeological wood conservation. Proc Natl Acad Sci.

[30] Walsh Z, Janeček E-R, Jones M, Scherman OA (2017). Natural polymers as alternative consolidants for the preservation of waterlogged archaeological wood. Stud Conserv.

[31] McHale E, Steindal CC, Kutzke H, Benneche T, Harding SE (2017). In situ polymerisation of isoeugenol as a green consolidation method for waterlogged archaeological wood. Sci Rep.

[32] Christensen M, Kutzke H, Hansen FK (2012). New materials used for the consolidation of archaeological wood–past attempts, present struggles, and future requirements. J Cult Herit.

[33] Broda M, Hill CAS (2021). Conservation of waterlogged wood—past, present and future perspectives. Forests.

[34] Babiński L (2015). Dimensional changes of waterlogged archaeological hardwoods pre-treated with aqueous mixtures of lactitol/trehalose and mannitol/trehalose before freeze-drying. J Cult Herit.

[35] Nguyen TD, Sakakibara K, Imai T, Tsujii Y, Kohdzuma Y, Sugiyama J (2018). Shrinkage and swelling behavior of archaeological waterlogged wood preserved with slightly crosslinked sodium polyacrylate. J Wood Sci.

[36] Nguyen TD, Wakiya S, Matsuda K, Ngoc BD, Sugiyama J, Kohdzuma Y (2018). Diffusion of chemicals into archaeological waterlogged hardwoods obtained from the Thang Long Imperial Citadel site, Vietnam. J Wood Sci.

[37] Imazu S, Ito K, Fujita H, Morgos A. The rapid trehalose conservation method for archaeological waterlogged wood and laquerware. In: Grant T. Cook C. editors. Proceedings of the 12th ICOM-CC Group on wet organic archaeological materials conference, Istanbul, 2013. Istanbul: Lulu.com; 2016. p. 110–7.

[38] Jensen P, Pedersen NB. Examination of D-mannitol as an impregnation agent for heavily degraded waterlogged archaeological wood. In: Grant T. Cook C. editors. Proceedings of the 12th ICOM-CC Group on wet organic archaeological materials conference, Istanbul, 2013. Istanbul: Lulu.com; 2016. p. 118–25.

[39] Broda M, Spear MJ, Curling SF, Ormondroyd GA (2021). The viscoelastic behaviour of waterlogged archaeological wood treated with methyltrimethoxysilane. Materials.

[40] Majka J, Zborowska M, Fejfer M, Waliszewska B, Olek W (2018). Dimensional stability and hygroscopic properties of PEG treated irregularly degraded waterlogged Scots pine wood. J Cult Herit.

[41] Vetter LD, den Bulcke JV, Acker JV (2010). Impact of organosilicon treatments on the wood-water relationship of solid wood. Holzforschung.

[42] Broda M, Mazela B (2017). Application of methyltrimethoxysilane to increase dimensional stability of waterlogged wood. J Cult Herit.

[43] Kilic M, Kilic AG. Kauramin tests for the Yenikapi shipwrecks. In: Grant T. Cook C. editors. Proceedings of the 12th ICOM-CC Group on wet organic archaeological materials conference, Istanbul, 2013. Istanbul: Lulu.com; 2016. p. 222–7.

[44] Babiński L (2007). Influence of pre-treatment on shrinkage of freeze-dried archaeological oak-wood. Acta Sci Pol Silv Colendar Rat Ind Lignar.

[45] www.rgzm.de/kur.

[46] Stelzner I (2017). Zur Nassholzkonservierung Bestimmung prozessrelevanter Eigenschaften für die Gefriertrocknung.

[47] Wittköpper M, Muskalla W, Stephan B, Le Boedec-Moesgard A, Gebhadt S, Klonk S. In: The KUR (conservation and restauration) project - a comparison of different methods to preserve waterlogged wood. Proceedings of the 12th ICOM-CC Group on wet organic archaeological materials conference, Istanbul, 2013. Istanbul: Lulu.com; 2016. p. 134–43.

[48] Cook C, Lafrance J, Li C. Preliminary assessment of a new PEG. In: Strætkvern, K. Williams. E. editors. Proceedings of th 11th ICOM-CC Group on wet organic archaeological materials conference, Greenville, 2010. Greenville: Lulu.com; 2010. p. 245–55.

[49] Cretté SA, Näsänen L, González-Pereyra NG, Rennison B (2013). Conservation and treatment monitoring of waterlogged archeological corks using supercritical CO_2_ and treatment monitoring using structured-light 3D scanning. J Supercrit Fluids.

[50] Schindelholz E, Blanchette RA, Held BW, Jurgens J, Cook D, Drews MJ, Hand S, Seifert B. An evaluation of supercritical drying and PEG/freeze drying of waterlogged archaeological wood. In: Straetkver K, Huisman DJ. editors. Proceedings of the 10th ICOM-CC Group on wet organic archaeological materials conference, Amsterdam, 2007. Amersfoort: Rijksdienst Voor Archeologie, Cultuurlandschap En Monumenten; 2009. p. 399–416.

[51] De Jong J (1977). Conservation techniques for old waterlogged wood from shipwrecks found in the Netherlands. Biodeterior Invest Tech.

[52] Stelzner I. Transfer into praxis. Evaluation of consolidants for freeze-drying archaeological wood. In: Williams E, Hocker E. editors. Proceedings of th 13th ICOM-CC Group on wet organic archaeological materials conference, Florence, 2016. Florence: Lulu.com; 2018. p. 325–32.

[53] Van Damme T, Auer J, Ditta M, Grabowski M, Couwenberg M (2020). The 3D annotated scans method: a new approach to ship timber recording. Herit Sci.

[54] Braovac S, McQueen CMA, Sahlstedt M, Kutzke H, Łucejko JJ, Klokkernes T (2018). Navigating conservation strategies: linking material research on alum-treated wood from the Oseberg collection to conservation decisions. Herit Sci.

[55] Kowalczuk J, Rachocki A, Broda M, Mazela B, Ormondroyd GA, Tritt-Goc J (2019). Conservation process of archaeological waterlogged wood studied by spectroscopy and gradient NMR methods. Wood Sci Technol.

[56] Bugani S, Modugno F, Łucejko JJ, Giachi G, Cagno S, Cloetens P (2009). Study on the impregnation of archaeological waterlogged wood with consolidation treatments using synchrotron radiation microtomography. Anal Bioanal Chem.

[57] Rankin K, Hazell Z, Middleton A, Mavrogordato M (2021). Micro-focus X-ray CT scanning of two rare wooden objects from the wreck of the London, and its application in heritage science and conservation. J Archaeol Sci.

[58] Wiesner I, Stelzner J, Million S, Kuhnt K, Bott K. The first wheels go round again. In: Grant T. Cook C. editors. Proceedings of the 12th ICOM-CC group on wet organic archaeological materials conference, Istanbul, 2013. Istanbul: Lulu.com; 2016. p. 197–8.

[59] Unger A, Planitzer J, Morgós A (1988). Röntgencomputer- und Magnetresonanztomographie zur Charakterisierung von archäologischem Naßholz. Holztechnologie.

[60] Demoulin T, Gebhard R, Schillinger B (2015). Neutron tomography of archaeological waterlogged wood. Restaur Archäol.

[61] Christensen M, Hansen FK, Kutzke H (2015). Phenol formaldehyde revisited-novolac resins for the treatment of degraded archaeological wood: novolac resins for treatment of degraded archaeological wood. Archaeometry.

[62] Stelzner I, Stelzner J, Martinez-Garcia J, Gwerder D, Wittkoepper M, Muskalla W, Egg M. Schuetz P. Non-destructive assessment of conserved archaeological wood using computed tomography. In: Bridgland J, editor. Transcending boundaries: integrated approaches to conservation. ICOM-CC 19th Triennial Conference preprints, Beijing, 2021. Paris: ICOM-CC; 2021; p. 1–11.

[63] Wittköpper M (1998). Der aktuelle Stand der Konservierung archäologischer Naßhölzer mit Melamin/Aminoharzen am Römisch-Germanischen Zentralmuseum. Arbeitsblätter für Restauratoren.

[64] Imazu S, Morgós A. An improvement on the Lactitol MC conservation method used for the conservation of archaeological waterlogged wood (The conservation method using Lactitol MC and Trehalose mixture). In: Hoffmann P, Spriggs JA, Grant T, Cook C, Recht A, editors. Proceedings of the 8th ICOM-CC Group on wet organic archaeological materials conference, Stockholm, 2001. Bremerhaven: ICOM-CC; 2002. p. 413–28.

[65] Smith CW (2003). Archaeological conservation using polymers: practical applications for organic artifact stabilization.

[66] Jensen P, Petersen AH, Straetkvern K, Ek M (2011). From the Skuldelev to the Roskilde ships—50 years of shipwreck conservation at the National Musem of Denmark. Shipwrecks 2011 proceedings, chemistry and preservation of waterlogged wooden shipwrecks, Stockholm, 2011.

[67] Cook C, Grattan D. A method of calculation the concentration of PEG for freeze-drying waterlogged wood. In: Hoffmann P, editor. Proceedings of the 4th ICOM-CC Group on wet organic archaeological materials conference, Bremerhaven, 1987. Bremerhaven: ICOM-CC; 1990. p. 239–52.

[68] Kellogg RM, Sastry CBR, Wellwood RW (1975). Relationships between cell-wall composition and cell-wall density. Wood Fiber Sci.

[69] Macchioni N, Pizzo B, Capretti C, Giachi G (2012). How an integrated diagnostic approach can help in a correct evaluation of the state of preservation of waterlogged archaeological wooden artefacts. J Archaeol Sci.

[70] Macchioni N, Pecoraro E, Pizzo B (2018). The measurement of maximum water content (MWC) on waterlogged archaeological wood: a comparison between three different methodologies. J Cult Herit.

[71] Brather S (2009). Zur Anwendung von Dichteangaben bei der Bestimmung der PEG-Tränkkonzentration mit dem PEGcon-Computerprogramm. Restaur Archäol.

[72] Feldkamp LA, Davis LC, Kress JW (1984). Practical cone-beam algorithm. J Opt Soc Am A.

[73] Stelzner J, Million S (2015). X-ray Computed Tomography for the anatomical and dendrochronological analysis of archaeological wood. J Archaeol Sci.

[74] Stamm AJ, Tarkow H (1947). Dimensional Stabilisation of Wood. J Phys Colloid Chem.

[75] Stamm AJ, Burr HK, Kline AL (1955). Heat-stabilized Wood (staybwood).

[76] Stamm AJ (1959). Effect of Polyethylene Glycol on the Dimensional Stability of Wood. For Prod J.

[77] Rowell RM, Youngs RL (1981). Dimensional stabilization of wood in use.

[78] Håfors B, Rowell RM, Barbour J (1990). The role of the wasa in the development of the polyethylene glycol preservation method. Archaeological wood; properties, chemistry and preservation.

[79] Wadell H (1935). Volume, shape and roundness of quartz particles. J Geol.

[80] de Jong J. The conservation of shipwrecks. In: ICOM-CC, editor. Preprints of the ICOM-CC 5th triennial meeting, Zagreb, 1978. Paris: ICOM-CC; 1978. p. 78/7/1–10.

[81] de Jong J. The conservation of waterlogged timber at Ketelhaven (Holland). In: ICOM-CC, editor. Preprints of the ICOM-CC 5th triennial meeting, Venice, 1975. Paris: ICOM-CC; 1975. p. 75/8/1–9.

[82] Mühlethaler B (1973). Conservation of waterlogged wood and wet leather.

[83] Jensen P, Jørgensen G, Schnell U. Dynamic LV-SEM analyses of freeze drying processes for waterlogged wood. In: Hoffmann P, Grant T, Spriggs JA, Cook C, Recht A, editors. Proceedings of the 8th ICOM-CC Group on wet organic archaeological materials conference, Stockholm, 2001. Bremerhaven: ICOM-CC; 2002. p. 319–33.

[84] Hoffmann P (2010). On the long-term visco-elastic behaviour of polyethylene glycol (PEG) impregnated archaeological oak wood. Holzforschung.

[85] Mietke H, Martin D. Sugar preservation of the Friesland ship. Chemical and microbiological investigations and insights. In: Bonnot-Diconne C, Hiron X, Khoi Tran Q, Hoffmann P, editors. Proceedings of the 7th ICOM-CC Group on wet organic archaeological materials conference, Grenoble, 1998. Grenoble: ICOM-CC; 1999. p. 204–9.

[86] Schiweck H (1998). Zucker/Saccharose, Seine anwendungstechnisch relevanten Eigenschaften bei der Nassholzkonservierung. Arbeitsblätter für Restauratoren.

[87] Spinella A, Chillura Martino DF, Saladino ML (2021). Solid state NMR investigation of the roman *Acqualadroni**rostrum*: tenth year assessment of the consolidation treatment of the wooden part. Cellulose.

[88] Cole-Hamilton DI, Kaye B, Chudek IA, Hunter G (1995). Nuclear magnetic resonance imaging of waterlogged wood. Stud Conserv.

[89] Mori M, Kuhara S, Kobayashi K, Suzuki S, Yamada M, Senoo A (2019). Non-destructive tree-ring measurements using a clinical 3T-MRI for archaeology. Dendrochronologia.

